# RANKL/RANK control *Brca1* mutation-driven mammary tumors

**DOI:** 10.1038/cr.2016.69

**Published:** 2016-05-31

**Authors:** Verena Sigl, Kwadwo Owusu-Boaitey, Purna A Joshi, Anoop Kavirayani, Gerald Wirnsberger, Maria Novatchkova, Ivona Kozieradzki, Daniel Schramek, Nnamdi Edokobi, Jerome Hersl, Aishia Sampson, Ashley Odai-Afotey, Conxi Lazaro, Eva Gonzalez-Suarez, Miguel A Pujana, for CIMBA, Holger Heyn, Enrique Vidal, Jennifer Cruickshank, Hal Berman, Renu Sarao, Melita Ticevic, Iris Uribesalgo, Luigi Tortola, Shuan Rao, Yen Tan, Georg Pfeiler, Eva YHP Lee, Zsuzsanna Bago-Horvath, Lukas Kenner, Helmuth Popper, Christian Singer, Rama Khokha, Laundette P Jones, Josef M Penninger

**Affiliations:** 1 IMBA, Institute of Molecular Biotechnology of the Austrian Academy of Sciences, Vienna 1030, Austria; 2 Department of Biological Sciences, University of Maryland-Baltimore County, Baltimore, MD 21250, USA; 3 Princess Margaret Cancer Centre, Toronto, Ontario, Canada M5G 1L7; 4 Lunenfeld-Tanenbaum Research Institute, Mount Sinai Hospital, 600 University Avenue, Toronto, Ontario, Canada M5G 1X5; 5 Department of Molecular Genetics, University of Toronto, Ontario, Canada M5S 3E1; 6 Department of Pharmacology, University of Maryland, Baltimore, School of Medicine, Baltimore, MD 21201, USA; 7 Department of Biological Sciences, Cornell University, Ithaca, NY 14853, USA; 8 Hereditary Cancer Program, Catalan Institute of Oncology, IDIBELL, L'Hospitalet de Llobregat, Barcelona, Catalonia, Spain; 9 Cancer Epigenetics and Biology Program, IDIBELL, L'Hospitalet de Llobregat, Barcelona, Catalonia, Spain; 10 ProCURE, Catalan Institute of Oncology, IDIBELL, L'Hospitalet de Llobregat, Barcelona, Catalonia, Spain; 11 Department of Public and Primary Care, Centre for Cancer Genetic Epidemiology, University of Cambridge, Cambridge, UK; 12 Centre for Genomic Regulation, The Barcelona Institute of Science and Technology, University Pompeu Fabra, Barcelona, Catalonia, Spain; 13 The Campbell Family Institute for Breast Cancer Research, University Health Network, Toronto, Ontario, Canada M5G 1Z5; 14 Departments of Obstetrics and Gynecology and Comprehensive Cancer Center, Medical University of Vienna, Vienna 1090, Austria; 15 Department of Biological Chemistry, School of Medicine, University of California, Irvine, CA 92697, USA; 16 Department of Experimental Pathology and Pathology of Laboratory Animals, Medical University Vienna and University of Veterinary Medicine Vienna, Vienna 1090, Austria; 17 Ludwig Boltzmann Institute for Cancer Research (LBI-CR), Vienna, Austria; 18 Research Unit Molecular Lung and Pleura Pathology, Institute of Pathology, Medical University Graz, Graz 8010, Austria

**Keywords:** *BRCA1*, RANK, RANKL, inherited breast cancer, mammary progenitor cells

## Abstract

Breast cancer is the most common female cancer, affecting approximately one in eight women during their life-time. Besides environmental triggers and hormones, inherited mutations in the *breast cancer 1* (*BRCA1*) or *BRCA2* genes markedly increase the risk for the development of breast cancer. Here, using two different mouse models, we show that genetic inactivation of the key osteoclast differentiation factor RANK in the mammary epithelium markedly delayed onset, reduced incidence, and attenuated progression of *Brca1;p53* mutation-driven mammary cancer. Long-term pharmacological inhibition of the RANK ligand RANKL in mice abolished the occurrence of *Brca1* mutation-driven pre-neoplastic lesions. Mechanistically, genetic inactivation of *Rank* or RANKL/RANK blockade impaired proliferation and expansion of both murine *Brca1;p53* mutant mammary stem cells and mammary progenitors from human *BRCA1* mutation carriers. In addition, genome variations within the *RANK* locus were significantly associated with risk of developing breast cancer in women with *BRCA1* mutations. Thus, RANKL/RANK control progenitor cell expansion and tumorigenesis in inherited breast cancer. These results present a viable strategy for the possible prevention of breast cancer in *BRCA1* mutant patients.

## Introduction

Risk factors for breast cancer development include exposure to environmental factors such as synthetic sex steroid hormones, endogenous hormones, or genetic predisposition. In particular, germline mutations in *breast cancer 1* (*BRCA1*) and *BRCA2* account for 2%-10% of breast cancer cases depending on the ethnic population^1^. Although BRCA1/2 are involved in the repair of double-strand breaks in DNA, human evidence suggests a relationship between *BRCA1/2* mutations, sex hormone levels, and cancer risk^2^. In addition, progesterone has been shown to play a role in mammary tumorigenesis of *Brca1/p53* mutant mice^3^. However, the molecular mediators of *BRCA1/2*-associated risk and sex steroid exposure have not been identified.

RANKL (receptor activator of NF-κB ligand), its receptor RANK, and the natural inhibitor osteoprotegerin (OPG) are essential for the development and activation of osteoclasts^4,5^. Based on these findings, RANKL inhibition with a monoclonal antibody has been successfully developed as a rational therapy against osteoporosis and skeletal-related events in cancer patients^6,7,8,9^. In addition, RANKL/RANK are essential for the formation of a lactating mammary gland during pregnancy^10^. RANKL is secreted by estrogen receptor (ER)- and progesterone receptor (PR)-positive mammary epithelial cells in response to progesterone, and subsequently acts in a paracrine fashion on ER/PR-negative epithelial progenitor cells, promoting proliferation and expansion of mammary epithelial cells^9,11,12^. Using genetic mouse models, we and others have previously shown that RANKL/RANK control progestin-driven mammary cancer^13,14^. Interestingly, RANK signaling acts on progenitor cells, which are also believed to be “seed cells” for triple-negative breast cancer in carriers with *BRCA1/2* mutations^15^. We therefore speculated that RANKL/RANK might have a role in the etiology of *BRCA1/2* mutation-driven breast cancer.

## Results

### Genetic inactivation of Rank protects from *Brca1* deletion-driven tumorigenesis

**Figure 1:**
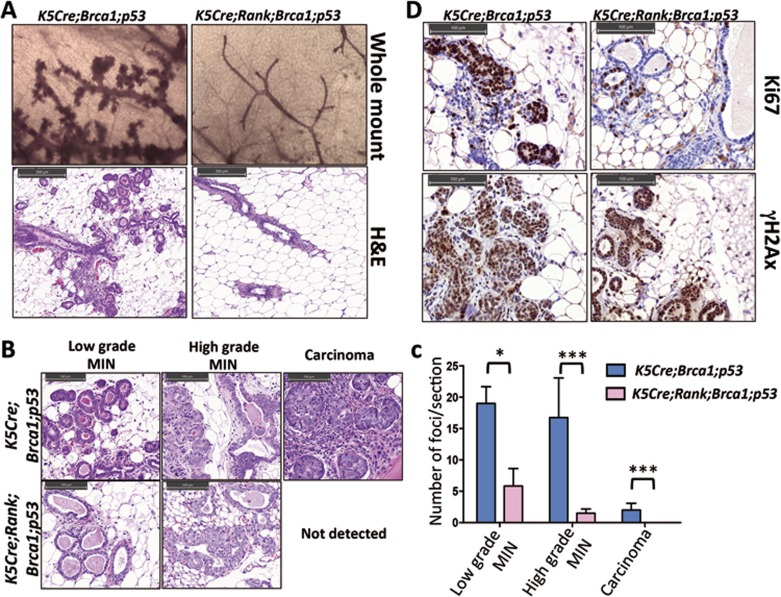
Ablation of *Rank* in mammary epithelial cells markedly decreases tumor formation in *Brca1/p53* mutant female mice. **(A)** Representative whole mount images (haematoxylin staining, magnification 52×) and paraffin sections (H&E staining, scale bar, 200 μm) of mammary glands from 4-month-old *K5Cre;Brca1;p53* double- and *K5Cre;Rank;Brca1;p53* triple-knockout littermate mice. **(B)** Representative images (H&E staining, scale bar, 100 μm) and **(C)** quantification of low-grade MINs, high-grade MINs and adenocarcinomas in mammary glands from 4-month-old *K5Cre;Brca1;p53* and *K5Cre;Rank;Brca1;p53* mutant littermates. Data are shown as average number of foci/section of 1 inguinal and 2 thoracic mammary glands per mouse +/− SEM. *n* ≥ 4 mice/group. ^*^P < 0.05, ^***^P < 0.001 (2-way ANOVA). **(D)** Representative images of Ki67 and γH2AX immunostaining of mammary glands from 4-month-old *K5Cre;Brca1;p53* double- and *K5Cre;Rank;Brca1;p53* triple-knockout littermates. Scale bar, 100 μm.

In mice the incidence of mammary tumors in the presence of only *Brca1* mutation is low. Therefore, to directly assess the role of RANKL/RANK in *Brca1* mutation-mediated tumorigenesis *in vivo*, we first deleted *Brca1* and *p53* in basal mammary epithelial cells and mammary progenitor cells using K5Cre mice^16^ to induce mammary cancer as previously reported^17,18^. This line was then crossed into *Rank^flox/flox^* mice to examine *Brca1* deletion-induced tumorigenesis in the presence or absence of RANK expression (Supplementary information, Figures S1A, S1B and S2). All mouse lines examined appeared to develop normal mammary glands at puberty. In 4-month-old control *K5Cre;Brca1;p53* double-mutant mice, we observed widespread epithelial hyperplasia (Figure 1A and Supplementary information, Figure S3) as well as low and high-grade mammary intraepithelial neoplasias (MINs) and invasive carcinomas (Figure 1B and 1C). The mammary glands of age-matched females with concomitant ablation of *Rank* appeared largely normal, displaying a significantly lower number of MINs and no detectable carcinomas (Figure 1A-1C and Supplementary information, Figure S3). Quantification of branching points in whole mount stainings from female littermates further showed that loss of RANK significantly decreased proliferation and pre-neoplasia observed in the absence of *Brca1* and *p53* (Supplementary information, Figure S1C). Enhanced proliferation of mammary epithelial cells in *K5Cre;Brca1;p53* double-mutant mice was confirmed using Ki67 immunostaining (Figure 1D and Supplementary information, Figure S1D). Importantly, we observed marked DNA damage in both double- and triple-mutant mice as determined by γH2AX immunostaining (Figure 1D and Supplementary information, Figure S1E). DNA damage was confirmed using a second marker, p53BP1 (Supplementary information, Figure S4A). Moreover, low- and high-grade MINs that developed in 4-month-old double- and triple-mutant mice expressed Cytokeratin5 (KRT5/CK5) and β-catenin, confirming the basal epithelial origin of these tumors (Supplementary information, Figure S4B and S4C). These data show that genetic deletion of *Rank* in basal mammary epithelial cells markedly abrogates the development of intraepithelial neoplasms and invasive carcinomas as a consequence of *Brca1;p53* mutations.

**Figure 2:**
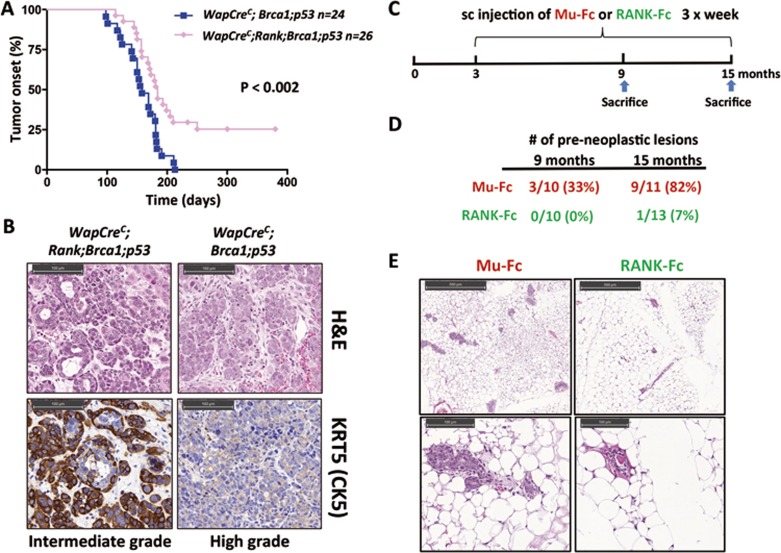
Genetic deletion or pharmacological inhibition of RANK reduces the incidence of *Brca1* deletion-driven mammary tumorigenesis. **(A)** Onset of palpable mammary tumors in *WapCre^C^;Brca1;p53* and *WapCre^C^;Rank;Brca1;p53* mice. Data are shown as percentage of tumor-free mice. **(B)** Representative histological sections of mammary tumors isolated from *WapCre^C^;Brca1;p53* and *WapCre^C^;Rank;Brca1;p53* mice showing different histological grades (H&E) and altered, grade-dependent KRT5/CK5 immunostainings. Scale bar, 100 μm. **(C)** Schematic of the regimen used to treat *MMTV-CreBrca1^flox11/flox11^* mice with control Mu-Fc or RANK-Fc to block RANKL/RANK *in vivo*. **(D)** Numbers and percentages of *MMTV-Cre Brca1^flox11/flox11^* mice with pre-neoplastic lesions that received subcutaneous (sc) injection of Mu-Fc or RANK-Fc for 6 or 12 months (3 times/week) and were sacrificed for analysis at 9 and 15 month of age. **(E)** Representative H&E-stained sections of mammary tissue of *MMTV-CreBrca1^flox11^* mice that received Mu-Fc or RANK-Fc and were sacrificed at 15 month of age. Magnification, 400×.

The occurrence of skin cancer commonly observed in the *K5Cre;Brca1;p53* double-^17,18^ as well as *K5Cre;Rank;Brca1;p53* triple-mutant mice precluded further analysis of mammary tumorigenesis beyond the 4 month time point. We therefore switched the Cre deleter line and introduced all three conditional alleles onto a WapCre^C^ mouse background^19^ (Supplementary information, Figure S5A). Of note, in this mouse line, the whey-acid protein (Wap) activity is independent of doxycycline and pregnancy, and the Cre activity is present in both luminal and basal epithelial cells in the mammary gland^19^ (Supplementary information, Figure S2). As expected from our previous work using whole-body *Rank* mutants or MMTVCre- and K5Cre-driven *Rank* deletion, WapCre^C^-mediated *Rank* deletion had no apparent effect on formation of the mammary gland during puberty (Supplementary information, Figure S5B). While all *WapCre^C^;Brca1;p53* mutant females developed palpable tumors starting around day 100 after birth, concomitant *Rank* deletion in the mammary epithelium significantly delayed tumor onset (Figure 2A). The median tumor onset for *WapCre^C^;Brca1;p53* mice was 158 days, whereas the median onset for *WapCre^C^;Rank;Brca1;p53* triple-mutant mice was 184 days. Importantly, while all *WapCre^C^;Brca1;p53* double-mutant females developed mammary carcinomas, 25% of *WapCre^C^;Rank;Brca1;p53* triple-mutant littermates never developed any tumors (Figure 2A). This was also reflected by the overall survival rates (Supplementary information, Figure S5C), even when we followed these females up to 2 years of age. Once mammary tumors developed in both double- and triple-mutant mice, their growth curves appeared to be similar (Supplementary information, Figure S5D). Thus, *WapCre^C^*-driven deletion of *Rank* delays the onset and in 25% of cases even completely prevents the development of *Brca1;p53* mutation-driven mammary cancer.

### Loss of RANK impairs tumor progression to high-grade malignancies

Histopathologic analysis revealed that double-knockout *WapCre^C^;Brca1;p53* mice feature high-grade tumors (60%) as well as intermediate-grade tumors (40%). These high-grade tumors typically exhibit more foci with higher degrees of anisocytosis and anisokaryosis, higher mitotic rates and fewer regions of glandular differentiation (Figure 2B and Supplementary information, Figure S6A). By contrast, triple-knockout mice never developed high-grade tumors but displayed intermediate-grade tumors characterized by low to intermediate anisocytosis and anisokaryosis, low to moderate mitotic rate and some foci of glandular differentiation (Figure 2B and Supplementary information, Figure S6A). In these tumors we observed effective deletion of *Rank*, *Brca1* and *p53* (Supplementary information, Figure S6B and S6C). All intermediate-grade tumors that developed in double- and triple-mutant mice expressed the basal epithelial marker CK5; however, in high-grade tumors from *WapCre^C^;Brca1;p53* double-mutant females we observed marked downregulation of CK5 expression (Figure 2B, Supplementary information, Figure S7A and S7B), supporting the notion of manifest epithelial dedifferentiation of these high-grade mammary cancers. Similar to CK5, we detected high expression of the basal myoepithelial marker p63 in *Rank* mutant tumors, whereas p63 expression was largely lost in the high-grade tumors from *WapCre^C^;Brca1;p53* double-mutant females (Supplementary information, Figure S7C). Tumor cells of both cohorts still expressed β-catenin, confirming the epithelial lineage, whereas ERα and PRs were not detectable (Supplementary information, Figure S7D-S7F), confirming that these tumors are hormone receptor negative. RNAseq profiling of mammary carcinomas from *WapCre^C^;Brca1;p53* and littermate *WapCre^C^;Rank;Brca1;p53* females showed differences in their molecular signatures (Supplementary information, Figure S8A and S8B; all primary data have been deposited to Gene Expression Omnibus reference GSE71362); hierarchical clustering of the Spearman's correlation showed similarity of our mouse tumors to previously defined basal-like mammary cancer^20^ (Supplementary information, Figure S8C). Thus, loss of RANK not only delays tumor onset, but also offsets progression to higher grades of malignancy.

Ki67 immunostaining of mammary tumors from *WapCre^C^;Brca1;p53* double-mutant and *WapCre^C^;Rank;Brca1;p53* triple-mutant females showed comparable proliferation of tumor cells in both genotypes (Supplementary information, Figure S9A and S9B), a finding that might explain that established tumors grow at a comparable “speed” (Supplementary information, Figure S5D). Immunostaining for γH2AX as well as p53BP1 showed that loss of *Brca1* and *p53* resulted in massive DNA damage in intermediate as well as high-grade tumors, irrespective of the presence or absence of RANK (Supplementary information, Figure S9C-S9F). Importantly, we also observed marked DNA damage in the mammary epithelium of a 2-year-old *WapCre^C^;Rank;Brca1;p53* triple-mutant female that had never developed any tumor (Supplementary information, Figure S10A). Although there was substantial DNA damage, the mammary glands displayed normal histology with sparse proliferative foci as determined by low Ki67 positivity (Supplementary information, Figure S10A and S10B). Analysis of mammary epithelial cells (MECs) isolated from the tumor-free 2-year-old *WapCre;Rank;Brca1;p53* female confirmed efficient deletion of *Brca1*, *p53* and *Rank* (Supplementary information, Figure S10C). These data show that loss of *Rank* protects mice from mammary tumorigenesis and tumor progression despite the presence of DNA damage due to the inactivation of *Brca1*.

### Pharmacological inhibition of RANKL prevents the development of *Brca1* mutation-driven pre-neoplastic mammary lesions

We next assessed whether, in addition to our genetic experiments, preventive pharmacological RANKL inhibition could also be used to mitigate tumor development in a *Brca1* mutant mouse background. To test this, we treated *MMTV-Cre;Brca1^flox11/flox11^* mice with RANK-Fc to block RANKL/RANK *in vivo* and as a control with Mu-Fc and followed the cohorts for up to 15 months (Figure 2C). We switched to this mouse model because these animals still express p53 and develop pre-neoplastic mammary lesions, which are thus driven solely by the loss of *Brca1*. Therefore, they better mimic the genetics of the human situation prior to the development of overt breast tumors, which is when a prophylactic would be used. 3/10 (33%) *MMTV-Cre;Brca1^flox11/flox11^* females developed pre-neoplastic lesions in the control group (Mu-Fc-treated). Intriguingly, not a single (0/10) *MMTV-Cre;Brca1^flox11/flox11^* female treated for 6 months with RANK-Fc developed pre-neoplastic lesions in the mammary gland. All tissues analyzed retained morphological characteristics resembling age-matched wild-type (WT) control females (Figure 2D and Supplementary information, Figure S11A). In mice treated for 12 month with control Mu-Fc, 9/11 (82%) females developed mammary lesions, while such lesions were only detectable in 1/13 (7%) females treated with RANK-Fc (Figure 2D and 2E). Further characterization of the mammary lesions by immunohistochemistry verified that these lesions were of epithelial origin (Supplementary information, Figure S11B). Moreover, we found that the pre-neoplastic lesions in Mu-Fc-treated mice did not express ERα or PRs, indicative of pre-neoplastic expansion of ERα- and PR-negative epithelial progenitors (Supplementary information, Figure S11C). These data show that pharmacological inhibition of RANKL can prevent the development of *Brca1* mutation-driven pre-neoplastic mammary lesions.

### RANK controls the expansion of *Brca1*-mutated mouse and human mammary progenitor cells

**Figure 3:**
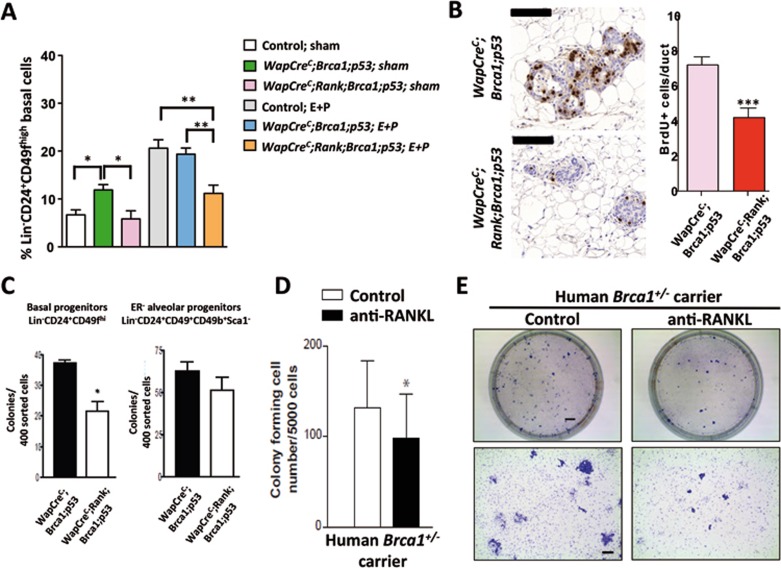
RANK mediates growth and expansion of *Brca1* mutant murine and *BRCA1* mutant human mammary progenitor cells. **(A)** Quantification of Lin^−^CD24^+^CD49f^hi^ basal mammary epithelial cells in ovariectomized WapCre-negative control, *WapCre^C^;Brca1;p53* double- and *WapCre^C^;Rank;Brca1;p53* triple-knockout mice treated with E+P or without hormone treatment (sham). Data are from 10-12-week-old mice, at which age we never observed any evident tumors, and represent mean +/− SEM (*n* ≥ 3 mice/group). ^*^P < 0.05, ^**^P < 0.005 (Student's *t*-test). **(B)** Representative histologic images and quantification of BrdU immunostaining of mammary glands of ovariectomized *WapCre^C^;Brca1;p53* double-knockout and *WapCre^C^;Rank;Brca1;p53* triple-knockout female mice treated with E+P showing reduced mammary epithelial proliferation (reduced BrdU positivity). Quantification data are shown as average numbers of BrdU^+^ cells per duct +/− SEM (*n* = 4 mice/cohort). ^***^P < 0.0001 (Student's *t*-test). Scale bar, 100 μm. **(C)** Colony forming capacity of basal progenitors and ER-negative alveolar mammary epithelial progenitor cells from *WapCre^C^;Rank;Brca1;p53* and *WapCre^C^;Brca1;p53* knockout mice. Data are shown as number of colonies per 400 sorted cells +/− SEM (*n* = 3 mice/group). ^*^P < 0.05, ns, not significant (Student's *t*-test). **(D**, **E)** Colony forming capacity (CFC) of human mammary progenitor cells. Human mammary progenitor epithelial cells were isolated from three women carrying heterozygous *BRCA1* mutations. Single mammary cell preparations were generated from organoids for CFC assays, plated, and were either untreated or treated with the anti-RANKL blocking Ab Denosumab (1 μg/ml). The quantification is shown in **D**. Data are shown as number of colonies per 5 000 plated cells +/− SEM (*n* = 3 different *BRCA1* carriers per group). ^*^P < 0.01 (Paired Student's *t*-test). Representative images of paired untreated control and anti-RANKL (Denosumab)-treated (1 μg/ml) mammary progenitor colonies are shown in **E**. Scale bars: upper panels, 5 mm; lower panels, 1mm.

Since genetic deletion or pharmacological inhibition of RANK delays the onset and even prevents *Brca1* and *Brca1;p53* mutation-driven mammary tumors, we speculated that RANKL/RANK might affect proliferation and expansion of mammary progenitor cells. To assess this hypothesis, we performed ovariectomy (ovx) on female mice followed by sham treatment (no hormones) or reconstitution with estrogen and progesterone (E+P). As previously reported^11,12^, sex hormone treatment resulted in a marked expansion of Lin^−^CD24^+^CD49f^hi^ basal mammary progenitor cells in both control as well as *WapCre^C^;Brca1;p53* double-mutant mice; however, this expansion was markedly reduced in the absence of RANK expression (Figure 3A and Supplementary information, Figure S12A). We also observed expansion of the basal Lin-CD24^+^CD49f^hi^ progenitor compartment in sham operated *WapCre;Brca1;p53* double-mutant mice, a phenotype that was again dependent on the expression of RANK (Figure 3A and Supplementary information, Figure S12A). Sex hormone-driven proliferation in ovx females was further determined by *in vivo* BrdU labelling. *WapCre^C^*-driven deletion of *Brca1* and *p53* resulted in markedly increased numbers of cycling mammary epithelial cells; this sex hormone-induced proliferation of mammary epithelial cells was significantly reduced in *Rank* mutant females (Figure 3B). Importantly, deletion of *Rank* significantly abrogated the *in vitro* colony formation capacity of basal Lin^−^CD24^+^CD49f^hi^ mammary progenitors from *WapCre^C^;Brca1;p53* mutant mice; the colony formation capacity within the ER^−^PR^−^Sca1^−^ alveolar progenitor compartment was also lower in the absence of RANK, albeit less pronounced (Figure 3C). Similar to our data using *WapCre^C^* deleter mice, sex hormone treatment resulted in expansion of the basal Lin^−^CD24^+^CD49f^hi^ progenitor compartment in ovarectomized *K5Cre;Brca1;p53* double-knockout mice as compared with sham operated mice, while this expansion was not observed in the absence of RANK (Supplementary information, Figure S12B). Thus, RANKL/RANK control sex hormone-induced expansion and activity of *Brca1;p53* mutant basal mammary progenitor cells. To determine the relevance of these results for the human disease, we isolated mammary progenitor cells from three women carriers of heterozygous *BRCA1* mutations who underwent prophylactic mastectomy, which is the standard of care. Importantly, *in vitro* colony-forming cell (CFC) assays performed on these human breast epithelial cells showed that RANKL inhibition using the clinically approved RANKL blocking antibody Denosumab significantly decreased the frequency of colony-forming cells (Figure 3D, 3E and Supplementary information, Figure S12C), indicating suppression of human mammary progenitor activity. These data reveal the capacity of RANKL inhibition to reduce the activity of mammary progenitor cells from women that carry germline *BRCA1* mutations.

### RANKL and RANK are highly expressed in pre-malignant lesions and breast cancer from human *BRCA* mutation carriers

**Figure 4:**
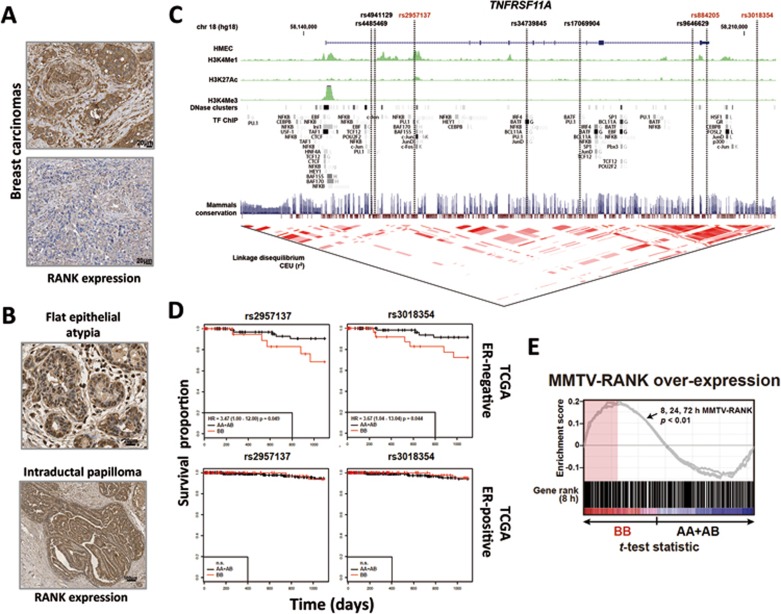
RANK expression in tumor tissues and *TNFRSF11A* variations linked to *BRCA1*-mutated status, risk of breast cancer, and cancer progression. **(A)** Representative images showing high and low RANK expression in invasive breast cancer tissues from *BRCA1* mutation carriers. **(B)** Representative examples of RANK expression in early epithelial pre-neoplastic lesions in women with *BRCA1* mutations. Representative images of a flat epithelial atypia and a typical intraductal papilloma are shown. Scale bars are shown. **(C)** Graph depicting the *TNFRSF11A* locus including the variants associated with breast cancer risk in *BRCA1* mutation carriers, and regulatory evidence from the ENCODE project and human mammary epithelial cells (HMECs). The rs884205 variant and the two in linkage disequilibrium and analyzed in TCGA dataset are marked in red. The exons are marked by blue-filled rectangles and the direction of transcription is marked by arrows in the gene structure. The chromosome 18 positions (base pairs (bp)) and linkage disequilibrium (*r*^2^) from a HapMap CEU panel are also shown. **(D)** Kaplan-Meier survival plots for the indicated *TNFRSF11A* genotypes (A and B represent the major and minor allele, respectively) in ER-negative and ER-positive breast tumors using TCGA data. **(E)** Gene Set Expression Analysis (GSEA) graphical output showing the positive correlation between the gene expression in tumors with the minor genotype (associated with poorer prognosis in ER-negative human breast cancer) and gene sets that characterize RANK overexpression in mammary epithelial cells of mice at 8, 24 or 72 h (GSE66174). All three sets were found to be significantly associated (*P* < 0.01); the score distribution curves overlap. The gene rank corresponds to the 8 h time point and the GSEA enrichment scores are shown^26^.

Following our observation that RANKL/RANK control *Brca1* mutation-driven tumorigenesis in mice and affect expansion of human *BRCA1^+/mut^* mammary progenitor cells, we analyzed RANK protein expression in human breast tumors that developed in *BRCA1* or *BRCA2* mutation carriers, and in non-*BRCA1/2* mutated individuals. RANK protein expression was absent or low (0 and 1+) in 74.5% of malignant breast tumors from *BRCA1/2* WT patients, with intermediate (2+) expression detectable in 25.5% of cases (Table 1). Notably, high RANK protein expression (3+) was not detected in any *BRCA1/2* WT patients. Conversely, intermediate or high levels of RANK were observed in 70.4% of *BRCA1*- and 80.7% of *BRCA2*-mutated tumors (Figure 4A and Table 1). A similar pattern was observed for RANKL protein expression which was detectable in 40.8% of tumors from *BRCA* WT patients, with intermediate or high expression in only 5.1% of breast cancer samples, but which was found in 59.1% of malignant tumors from *BRCA1* germline mutation carriers with intermediate or high expression in 14.8% samples. Similarly, 69.6% of tumors from *BRCA2* mutation carriers expressed RANKL with intermediate or high expression in 12.5% samples (Table 1). There was also a highly significant correlation between tumor grade and RANK protein expression in the overall series. While low-grade (G1) tumors expressed intermediate or high RANK levels in 28% of cases, 50.8% of grade 2 (G2) and 73.3% of all grade 3 (G3) tumors exhibited intermediate or high RANK protein expression (*P* < 0.001) (Supplementary information, Figure S12D and Table S1). RANKL protein staining was detected in 41.7% of G1 tumors, 56.3% of G2 tumors, and 51.2% of G3 tumors and there was no significant difference in respect to grading (*P* = 0.47) (Supplementary information, Figure S12D and Table S1). We next analyzed whether RANK expression was already present in the earliest tumor lesions detectable in the breast tissue of *BRCA1* mutation carriers. In all cases of such early lesions analyzed (*n* = 11 patients) including flat epithelial atypia and intraductal papillomas we detected high RANK expression (Figure 4B). Thus, RANK is highly expressed in pre-malignant lesions as well as in breast cancer that has developed in *BRCA1* and *BRCA2* mutation carriers.

### Common variants in RANK are associated with increased breast cancer risk in human *BRCA* mutation carriers

Since RANK/RANKL critically influence *Brca1* mutation-driven breast carcinogenesis in mice, we assessed the role of genetic modifiers of breast cancer risk in women with inherited *BRCA1* mutations at the corresponding locus encoding for human RANK, *TNFRSF11A*. This analysis was performed using data from the Collaborative Oncological Gene-environment Study (iCOGS) that included 51 *TNFRSF11A* single-nucleotide polymorphisms (SNPs) genotyped in ∼15 200 *BRCA1* and ∼8 200 *BRCA2* mutation carriers^21,22^ (Supplementary information, Table S2). Using a retrospective likelihood approach, we identified six SNPs that were significantly associated with breast cancer risk in the overall series of *BRCA1* mutation carriers and/or in ER-negative or triple-negative subtypes (Figure 4C, Table 2 and Supplementary information, Table S2). In addition, we found two SNPs in *TNFRSF11A* significantly associated (*P* < 0.05) with breast cancer risk in *BRCA2* mutation carriers (Supplementary information, Table S3). Interestingly, the rs884205 change (C→A) introduces an ATTAAA motif in the 3′-untranslated (3′-UTR) region of *TNFRSF11A*, providing a poly(A) signal for the nearby alternative polyA site (PolyA_DB Hs.204044.1.8; PolyA-seq GSE30198). Of note, the minor A allele, which we find predicts increased risk of breast cancer, correlates with enhanced RANK expression in various tissues using GTEX analysis, and the same allele has been previously associated with decreased bone density^23,24^, consistent with an over-activation of RANK-mediated signaling. Moreover, the G allele of rs4485469 was found to be associated with a reduced risk of breast cancer in the *BRCA1* mutation carriers (Figure 4C, Supplementary information, Tables S2 and S3), and we again find increased RANK expression linked to the complementary allele (A) in GTEX data. To assess these observations further, we analyzed data from The Cancer Genome Atlas (TCGA)^25^. While rs884205 was not present in TCGA, it was partially correlated (*r*^2^ ≈ 0.45) with two included variants, rs2957137 and rs3018354; a survival analysis using the recessive model for these variants revealed a significant association in ER-negative, but not in ER-positive breast cancer (Figure 4D). These two SNPs were not genotyped in iCOGS, but their imputed results (*r*^2^ > 0.94) again revealed significant associations (*P* = 0.021) with breast cancer risk in *BRCA1* mutation carriers, with effect estimations similar to rs884205: rs2957137 HR = 1.079, rs3018354 HR = 1.052. Moreover, expression analyses revealed that the ER-negative tumors with poorer prognosis have activated RANK signaling, as shown by their positive expression correlation with time series signatures of RANK overexpression^26^ (Figure 4E). These data indicate that common variations in *TNFRSF11A* modify the risk of developing breast cancer in *BRCA* mutation carriers.

## Discussion

RANKL acts in a paracrine fashion on the membranous RANK of ER/PR-negative epithelial cells of the breast to expand the stem and progenitor cell populations^27,28^. Our data show that RANKL/RANK also regulate the expansion and functional capacity of progenitor cells on a *Brca1/p53* mutant background and, most importantly, on mammary stem cells from women with heterozygous germline *BRCA1* mutations. Since RANKL is induced by progesterone, these data could explain why sex hormones, in particular progesterone, play an important role in *BRCA1* mutation-driven tumorigenesis in mouse models and humans. Studies have also demonstrated that *Brca1* deficiency increases the activity of E+P signaling pathways during mammary gland development and promotes mammary epithelial cell growth^29,30,31^. In particular, loss of full-length *Brca1* exaggerates the mammary growth in response to exogenous progesterone in virgin mice^30^. The importance of PR signaling in mutant *Brca1* tumorigenesis was further demonstrated by Poole *et al*.^3^ who showed that tumor development in *Brca1/p53* deficient mice could be prevented by the PR antagonist RU-486. Together, these findings highlight the potential value for RANKL inhibition in *BRCA1*-associated cancers at the early stages of tumorigenesis. Whether RANKL inhibition has an advantage over Tamoxifen and/or oophorectomy needs to be demonstrated in future experiments, especially in careful, clinical trials.

We also report, analyzing iCOGS data for more than 23 000 women with germline *BRCA1/2* mutations, that common variation in *TNFRSF11A*, linked to altered *RANK* expression, may be associated with breast cancer risk in *BRCA1* mutation carriers and, furthermore, the survival of patients with ER-negative tumors. Some of these identified polymorphisms are directly associated with altered gene expression and function of RANK. Further fine-scale mapping genetic analyses and studies on the regulation of RANK expression, and also expression of RANKL and the natural RANK inhibitor OPG, are warranted, since the 3′-UTR of RANK can be additionally regulated by various microRNAs, some of which have been directly associated with human breast cancer^32^. Since *BRCA1* mutation carriers are at high risk of ovarian cancer, it will be important to also explore the role of RANKL/RANK blockade in ovarian carcinogenesis. Most importantly, our work here shows that pharmacological inhibition of RANKL almost completely prevents the development of pre-neoplastic lesions due to a *Brca1* mutation. Our findings therefore raise the possibility that inhibition of RANKL, for which there is an already approved drug with a good safety record, could offer a novel, targeted approach for breast cancer prevention in *BRCA1* mutation carriers.

## Materials and Methods

### Mice

*Rank^floxed^* (*Tnfrsf11*^tm1.1Pngr^) and *WapCre^C^;Brca1^floxed^;p53^floxed^* (*Tg-WapCre^C^*, *Brca1*^tm2Cxd^, and *Trp53*^tm1Elee^) knockout mice have been previously generated in our laboratories^3,16^. K5Cre (*Tg-KRT5-Cre*) mice were purchased from the Jackson Laboratory. Of note, in our cohorts, the control mice examined were either Cre-negative but carried the *Brca1^flox^;p53^flox^*, and/or *Rank^flox^* alleles or were K5Cre positive in the absence of dual floxed alleles; none of these controls examined showed any epithelial hyperplasia in the mammary glands. *Brca1* conditional knockout mice with two exon 11 floxed *Brca1* alleles (*Brca1^flox11/flox11^*) carrying the mouse mammary tumor virus (MMTV)-Cre recombinase gene (*MMTVCre;Brca1^flox11/flox11^*) were maintained on C57Bl/6 genetic background. The *Rosa26^eYPF^* reporter mouse line has been previously reported^33^. Animals were genotyped by PCR analysis of genomic DNA. Mice were maintained in temperature-controlled conditions. All animal experiments were carried out in agreement with the ethical animal license protocol in accordance with the current laws of Austria and in accordance with institutional guidelines approved by the University of Maryland, Baltimore Animal Care and Use Committee.

### Histology and immunohistochemistry

Mouse tissue samples were fixed in 4% paraformaldehyde (PFA) overnight at 4°C and embedded in paraffin after dehydration in ascending concentrations of ethanol. For histological analysis, 2-4 μm-thick paraffin sections were prepared and stained with haematoxylin and eosin. For immunohistochemistry, after routine processing and antigen retrieval procedures, the following antibodies were used. Paraffin sections were stained using previously described protocols^34^ with antibodies against: KRT/CK5 (1:1 000, Rabbit anti-human KRT5, Sigma Aldrich, SAB4501651), γH2AX (1:500, Rabbit anti-human H2AFX, ph-Ser139, Novus Biologicals, NB100-79967), TP53BP1 (1:200, Rabbit anti-human TP53BP1 (E247), Bethyl Laboratories, IHC-00001), Ki67 (1:1 000, Rabbit anti-human Ki67, Novocastra, NCL-Ki67p), Ki67 (1:500, Rat anti-mouse Ki67, Affymetrix E-bioscience, 14-5698), BrdU (1:200, Rat anti-BrdU (BU1/75(ICR1)), Abcam, ab6326), CTNNB1 (1:250, Rabbit anti-CTNNB1 (E247), Abcam, ab32572). CASP3, Cleaved (1:200, Rabbit anti-human Cleaved Caspase 3, Cell Signalling, 9661), ERα (1:100, Rabbit anti-mouse ERα, Santa Cruz, sc542), PR (1:100, Rabbit anti-human PR, Santa Cruz, sc538), TP63 (1:150, Mouse anti-human TP63, Santa Cruz, sc8341), CD265/RANK-patient samples (1:1 000, Mouse anti-human CD265 (9A725), Acris Antibodies, TA336373), and CD254/RANKL ― patient samples (1:1 000, anti-human CD254, Acris Antibodies, ID8600).

Histomorphometric indices were calculated as the number of positive epithelial cells divided by the total number of epithelial cells, with no fewer than 1 000 counted nuclei for Ki67 and γH2AX staining. Mammary epithelial neoplasias (MINs) and adenocarcinomas in histologic sections were designated and evaluated in accordance with predefined criteria proposed by the Mouse Models of Human Cancers Consortium and INHAND^35,36^. Briefly, low-grade MINs were defined by the presence of ducts with intact basement membrane, at least one layer of atypical cells, hyperchromatic nuclei, luminal and/or myoepithelial cells with little cytoplasm, at least one layer and an increased mitotic rate. High-grade MIN lesions were defined by less organized glandular patterns, increased layers of epithelium, pleomorphism of nuclei and/or epithelial cells and/or an increase in mitotic figures with an intact basement membrane. Carcinomas were defined by an invasive growth pattern, increased cellular and nuclear pleomorphism and transgression of the basement membrane. The extent of KRT5 and TP53BP1 immunopositivity was quantified in mouse mammary lesions with *Definiens Tissue Studio*TM software. The software algorithm was trained by manual delineation and classification of representative areas in representative slides to identify positive targets and exclude non-targets and to create a quantification solution. Target regions were identified on the basis of morphology and positive immunohistochemical (IHC) staining. Subsequently the solution was applied to the digital slide sets to obtain automated quantification results. The validity of the analysis was confirmed by histopathologic verification of representative slides from both groups. For imaging and digital quantification, stained slides were scanned using a Pannoramic slide scanner (3D Histech).

For murine Rank immunostainings on frozen samples, tissue samples were snap frozen in OCT and 5-10-μm-thick frozen sections were prepared. After drying at room temperature, sections were fixed in Aceton at −20 °C for 5-10 min. Samples were first blocked for 45 min in freshly prepared H_2_O_2_ (0.3%), followed by blocking in TNB buffer for 30 min (Perkin Elmer, TSA fluorescein amplification kit) and Avidin and Biotin block for 15 min respectively (Avidin/Biotin Blocking kit, Vector Lab). Samples were incubated with a primary biotinylated anti-mRANK antibody (BAF692, R&D, 1:50) overnight at 4 °C followed by incubation with Streptavidin-HRP (1:100) for 30 min at room temperature and incubation with the working solution for 5-10 min. Samples were mounted with DAPI. For analysis of mammary glands after RANK-Fc treatment, the right inguinal mammary gland from each mouse was fixed in 10% buffered formalin (Fisher Scientific, Pittsburgh, PA) overnight at 4 °C and embedded in paraffin using standard techniques for H&E staining and IHC analysis. ERα IHC was performed on formalin-fixed, paraffin-embedded tissue sections using mouse on mouse (M.O.M) peroxidase kit (PK-2200, Vector Laboratories Inc., Burlingame, CA, USA) as previously described^37^ using a 1:25 dilution of the ERα antibody (M7047, Dako, Carpinteria, CA, USA) for 30 min. Polyclonal Rabbit Anti-Human Progesterone Receptor, Dako, A0098). Digital photographs were taken using a Nikon 50i Upright Microscope System with a high Resolution 5 Megapixel Color Digital Camera system (Nikon Instruments Inc., Melville, NY, USA). All histomorphology and immunohistochemistry of the subsets of tissues were qualitatively or semiquantitatively evaluated by board certified pathologists (AK, HP, LK, ZBH).

### Whole mount stainings

Mammary glands were dissected, placed on a microscope slide and fixed in 4% PFA overnight. To remove the adipose tissue, mammary glands were incubated in acetone for 3 h followed by rehydration in 100% ethanol and 95% ethanol for 1 h, respectively. Mammary glands were stained with hematoxylin for 3 h and afterwards incubated in slightly basic tap water for 2 h. Excessive hematoxylin was removed by destaining the glands in 50% ethanol acidified with 25 ml 1.0 M HCl/liter. After dehydration in 70%, 95%, and 100% ethanol, respectively, mammary glands were stored in xylene.

### Ovariectomy and hormone treatments

Six to eight-week-old female mice were anesthetized by intraperitoneal injections of Ketasol (5 mg/ml) and Xylasol (0.8 mg/ml). Bilateral small incisions were made under sterile conditions to open the peritoneal cavity. Ovaries, including the surrounding adipose tissue, were excised. Mice were allowed to recover for at least 2 weeks before further experiments were performed. For hormone supplementation mice were implanted subcutaneously on the right flank with slow release pellets containing a combination of progesterone and 17β-estradiol (Innovative Research of America, 21 day release, 0.14 mg 17β-estradiol + 14 mg progesterone).

### qRT-PCR

Total RNA of tumors and isolated mammary epithelial cells was prepared using the RNeasy Mini Kit (Qiagen), according to the manufacturer's instructions. cDNA synthesis was performed using the iScript cDNA synthesis kit (Bio-Rad). For qRT-PCR the following primers were used:

β-actin forward primer: 5′-GCTCATAGCTCTTCTCCAGGG-3′

β-actin reverse primer: 5′-CCTGAACCCTAAGGCCAACCG-3′.

Rank forward primer: 5′-CCCAGGAGAGGCATTATGAG-3′

Rank reverse primer: 5′-CAGCACTCGCAGTCTGAGTT-3′

Brca1 forward primer: 5′-TAAGCCAGGTGATTGCAGTG-3′

Brca1 reverse primer: 5′-TGCCCTCAGAAAACTCACAA-3′

P53 forward primer: 5′-TGGAAGACAGGCAGACTTTTC-3′

P53 reverse primer 5′-CCCCATGCAGGAGCTATTAC-3′

### Mammary epithelial cell isolation

Mammary epithelial cells were isolated as previously described^13^. Briefly, mice were sacrificed and mammary fat pads were removed using sterile dissection instruments. Mammary glands were transferred in 50 ml Falcon tubes and incubated in complete EpiCult medium (EpiCult-B basal medium, EpiCult-B proliferation supplements, 10 ng/ml rh bFGF, 10 ng/ml rh EGF, 4 μg/ml Heparin and 5% FCS) and 2.5× collagenase/hyaluronidase at 37 °C in a shaking incubator for 2.5 h. Red blood cell lysis was performed using ammonium chloride. Pellets were consecutively treated with 0.25% trypsin-EDTA and prewarmed dispase containing 200 μl of 1 mg/ml DNase. After filtering single mammary epithelial cells were prepared for FACS analysis or lysed for RNA purification.

### Flow cytometry

Multiparameter flow cytometry analyses were performed on mammary epithelial single-cell suspensions by immunostaining for 20 min at 4 °C in FACS buffer (PBS, 2% FCS, 2 mM EDTA). Before adding the fluorescently labeled antibodies, Fc-receptors were blocked with anti-CD16/CD32 (Pharmingen) antibodies was performed. Mammary epithelial cells were labeled with antibodies directed against CD24 (Pharmingen #553261), CD49f (Pharmingen #551129) and CD61 (eBioscience #11-0622-82). Non-epithelial cells were excluded using antibodies directed against CD45 (BD #553078), CD31 (BioLegend #102404) and Ter119 (BioLegend #116204). Flow cytometry data were acquired using a Fortessa LSRII flow cytometer (FACSFortessa^™^, BD), equipped with FacsDiva^™^ software (BD). Data analysis was performed using FlowJo^™^ software (Tree Star).

### Bromodesoxyuridin (BrdU) labeling

For BrdU pulse labeling, ovariectomized mice were stimulated with 17β-estradiol and progesterone for 2 weeks. BrdU (0.4 mg/g body weight) was injected intraperitoneally. Mice were sacrificed 2 h later and tissues were harvested.

### Human breast epithelial preparations

Human breast tissue samples were obtained from disease-free pre-menopausal women undergoing reduction mammoplasty or prophylactic mastectomy (*BRCA1* mutation carriers) with informed patient consent and Institutional Research Ethics Board approval from St. Michael's Hospital and from the University Health Network. Single-cell suspensions were prepared from previously isolated and cryopreserved organoids as previously described^38^.

### Murine and human mammary colony-formation assay

Mouse mammary epithelial subsets were FACS-sorted from individual mice and plated with irradiated fibroblasts in DMEM:F12 (3:1) medium containing 10% FBS, insulin (Life Technologies), cholera toxin (Sigma), adenine (Sigma), hydrocortisone (STEMCELL Technologies), and Rock inhibitor (Reagents Direct) and cultured in 5% oxygen conditions. Colonies were scored after 7-10 days. For human CFC assay, mammary epithelial progenitors were isolated from *Brca1* heterozygous mutation carriers undergoing pre-emptive surgery, and assays were performed in dishes precoated with a thin layer of collagen (STEMCELL technologies) in complete Epicult-B medium (STEMCELL Technologies) with irradiated feeders as previously documented^39^ and cultured in 5% oxygen conditions.

### Bioinformatics

PolyA-mRNA of *WapCre^C^;Brca1;p53*, and *WapCre^C^;Rank;Brca1;p53* mammary tumors was isolated and the generated libraries were sequenced by 50-bp single-end Illumina mRNA sequencing. Reads were aligned using Tophat v2.0.10 and bowtie v0.12.9, FPKM estimation was performed with Cufflinks v2.1.1, aligned reads were counted with HTSeq v0.6.1p1, and differential expression analysis was performed with DESeq2 v1.6.2. Significantly differentially expressed (DE) genes were selected with a false discovery rate (FDR) of 5%. Pathway enrichment was evaluated using GSEA against the Molecular Signature Database MSigDB v5 gene-set collection. For GSEA, human to mouse conversion of official gene symbols was performed using HomoloGene ortholog assignment. Gene sets with significant enrichment were selected based on a FDR *q*-value cut-off of 1%. RNAseq data and GSEA analysis data have been deposited to the NCBI Expression Omnibus and all data are accessible through the GEO accession number GSE71362 (NCBI tracking system #17490198). Briefly, gene set enrichment analysis (GSEA) using KEGG comparing mammary tumors growing in *WapCre^C^;Brca1;p53* double- vs *WapCre^C^;Rank;Brca1;p53* triple-mutant mice showed significantly enhanced expression of genes annotated to Basal Cell Carcinoma, Notch signaling, Hedgehog signaling, and metabolism of xenobiotics by cytochrome P450 in the triple-mutant tumors, whereas in double-mutant tumors genes annotated to aminoacyl tRNA biosynthesis, RNA degradation, and Citrate/TCA cycle were higher expressed. Using Hallmark GSEA analysis, we found enhanced expression of genes annotated to Wnt-catenin signaling, Notch signaling, the p53 pathway, late estrogen response, and Ras signaling in *Rank*-deficient tumors, whereas genes annotated to Myc and E2F targets, mTORC1 signaling, G2M checkpoint and mitotic spindle, inflammation and allograft rejection, as well as the unfolded protein response (UPR) were significantly upregulated in the double-knockout tumors. Of note, the UPR has recently been associated with triple-negative breast cancer^40^, aminoacyl-tRNA synthetases have been linked to tumorigenesis^41^, and multiple cross-talks have been established between the Wnt and RANKL/RANK pathways, including molecular cross-talks that control mammary progenitor cells^11,42^. Thus, *Rank* inactivation in a *WapCre^C^;Brca1;p53* mutant background results in mammary tumors that exhibit expression differences in genes annotated to oncogenesis, basic metabolism, RNA metabolism, or the regulation of mammary stem cells. The GSEA tool was run using default values for all parameters and using the *t*-statistic of the difference of gene expression between ER-negative tumors with the AA+AB and BB genotypes. We used transcriptome data to compare our mouse model with murine data previously assigned to mouse breast-cancer subtypes and linked to human subtypes^20,43^. Log expression estimates of our and published mouse models (NCBI GEO GSE3165) were combined by adjusting for the batch effect using Combat^44^. Hierarchical clustering of the Spearman correlation of samples shows similarity of our mouse model to basal-like samples. No consistent classification could be obtained using TNBCtype, a tool designed to identify potential Triple-negative breast cancer (TNBC) subtype membership^45,46^ (applied to log2FPKM expression data from our mouse model after mapping the data to human orthologous identifiers using HomoloGene).

### RANK-Fc treatment

Three-month-old *MMTV-Cre;Brca1^flox11/flox11^* mutant were treated with RANK-Fc (10 mg/kg three times/week; *n* = 26, RANK-Fc was provided by Amgen Pharmaceuticals, Inc.) subcutaneous injection (s.c.) or mouse Fc-fragment (Mu-Fc; 10 mg/kg three times/week, s.c. as a control; *n* = 22). The animal weights were monitored weekly. The treatment regime was well tolerated by all the mice and the weights were stably maintained throughout the length of treatment. Only one mouse in the Mu-Fc 15 months cohort (control) group was found dead in the cage due to unknown causes. Mammary tumor formation was determined by palpation and confirmed by histologic examination. Cohorts of mice from each experimental group were euthanized at 9 and 15 months of age. At necropsy, all ten mammary glands were exposed following a midline incision and glands were inspected visually, palpated, and digital photographs taken. Mammary glands were collected and processed for whole mount, hematoxylin and eosin (H&E) and IHC analyses, or snap frozen in liquid nitrogen and stored at −80 °C for gene and/or protein expression.

### Human breast cancer samples

Tumor samples were provided as formalin fixed, paraffin embedded tissues by the Center for Familial Breast and Ovarian Cancer at Medical University of Vienna and by the Kathleen Cunningham Foundation Consortium for research into Familial Breast cancer. The analysis was approved by the local Institutional Review Board-IRB: the Ethikkommissionsbescheid (IRB) number which covers the analyses is EK 056/2005, Amendment Vers. 1.0 of 4.12.2014 “Modifiers des Krebsrisikos in BRCA1/2 Mutationsträgerinnen”. Data were analyzed using SPSS Ver21.0 (Chicago, IL, USA). Women diagnosed with breast cancer who had undergone surgeries and gave consent to germline mutation testing were included in this analysis. All samples, including the *BRCA1/2* WT samples, were confirmed by sequencing. *χ*^2^-test and Fisher's Exact tests were used to compare the difference within RANK and RANKL categories. Samples classified as unknown for any variables were excluded from analyses. Two-sided *P* values ≤ 0.05 were considered to be statistically significant.

### Human cohort studies

This analysis was performed using data from the Collaborative Oncological Gene-environment Study (iCOGS) that included 51 *TNFRSF11A* (encoding for RANK) SNPs genotyped in ∼15 200 *BRCA1* and ∼8 200 *BRCA2* mutation carriers. The iCOGS design, quality controls, and statistical analyses have been previously described^21,22^. The TCGA genotype data from primary breast tumors were obtained following an approved request and through the data portal (https://tcga-data.nci.nih.gov/tcga). The survival analysis as a function of the genotypes was performed using the Cox proportional hazards regression and the significance of the association assessed using the log-rank test.

### Statistics

All values in the paper are given as means ± sem. Comparisons between groups were made by Student's *t*-test or 2-way ANOVA using GraphPad Prism (GraphPad Software, San Diego, CA, USA) or R statistical software. For the Kaplan–Meier analysis of tumor onset and survival a log-rank test was performed. *P* < 0.05 was accepted as statistically significant.

## Author Contributions

VS performed most of the experiments in RANK mutant mice with help from IK, DS, RS, MT, IU, LT and SR. GW performed lineage tracing experiments. KOB performed majority of the RANK-Fc treatment experiments and data analysis. JH and AS administered RANK-Fc and Mu-Fc treatments and data analysis. NE and AOA contributed to the ER and PR IHC experiments and data analysis. PAJ and RK analyzed and FACS-sorted mammary epithelial populations for colony forming assays and performed the human CFC assays. JC and HB provided human mammary gland epithelial cells. MN performed all bioinformatics analysis. AK, ZBH, and LK are board certified pathologists and performed histology and immunohistology assessments. MAP, EV and HH provided and analyzed TCGA data, CL, EGS and MAP analyzed and provided human data for CIMBA. HP performed immunostainings on human breast cancer samples provided by YT and CS. EYHPL provided BRCA1 and p53 mutant mice. LPJ directed and designed the RANK-Fc treatment experiments. JMP coordinated the project and wrote the manuscript.

## Competing Financial Interests

IMBA has applied for a patent on using RANKL inhibition to block breast cancer. Amgen, Inc. provided RANK-Fc and partial financial support (LPJ) for this study.

## Figures and Tables

**Table 1:** RANKL and RANK protein expression in *BRCA1* mutant, *BRCA2* mutant, and *BRCA1/2* WT human breast tumors.

Intensity	RANK^1^						RANKL^1^					
	WT		*BRCA1^2^*		*BRCA2^3^*		WT		*BRCA1^2^*		*BRCA2^3^*	
	(*n* = 98)		(*n* = 88)		(*n* = 62)		(*n* = 98)		(*n* = 88)		(*n* = 56)	
	N	%	N	%	N	%	N	%	N	%	N	%
0	0	0	2	2.3	0	0	58	59.2	36	40.9	17	30.4
1+	73	74.5	24	27.3	12	19.4	35	35.7	39	44.3	32	57.1
2+	25	25.5	34	38.6	30	48.4	5	5.1	13	14.8	7	12.5
3+	0	0	28	31.8	20	32.3	0	0	0	0	0	0

^1^Column percentages presented.

^2^Includes obligate carriers: *BRCA1*, *n* = 15.

^3^Includes obligate carriers: *BRCA2*, *n* = 2.

RANK and RANKL expression levels were determined by immunohistochemistry by board certified pathologists. In all cases, the *BRCA1* and *BRCA2* mutation status was determined by sequencing. *P* values (χ^2^ or Fisher's Exact test): RANK (0/1+ vs 2+/3+): WT vs *BRCA1/2* mutations, *P* < 0.001; WT vs *BRCA1*, *P* < 0.001; WT vs *BRCA2*, *P* < 0.001; *BRCA1* vs *BRCA2*, *P* = 0.158. RANKL (0 vs 1+/2+/3+): WT vs *BRCA1/2* mutations, *P* < 0.004; WT vs *BRCA1*, *P* = 0.013; WT vs *BRCA2*, *P* = 0.001; *BRCA1* vs *BRCA2*, *P* = 0.201.

**Table 2:** *TNFRSF11A* genotyped iCOGS variants associated (*P* < 0.05) with breast cancer risk in BRCA1 mutation carriers and/or ER-negative or overall triple-negative subtypes.

SNP ID	All *BRCA1*-mutated		ER-negative		Triple-negative		rs884205 (r2)
	HR (95% CI)	*P* value	HR (95% CI)	*P* value	HR (95% CI)	*P* value	
rs9646629	1.052 (1.006-1.096)	2.15E-02	1.061 (1.008-1.116)	2.20E-02	1.072 (1.008-1.140)	2.60E-02	0.54
rs4485469	0.956 (0.918-0.996)	3.55E-02	0.944 (0.899-0.992)	2.10E-02	0.943 (0.889-1.001)	5.40E-02	0.06
rs34739845	0.933 (0.876-0.999)	4.07E-02	0.9154 (0.847-0.990)	2.70E-02	0.907 (0.826-0.997)	4.30E-02	0.003
rs4941129	1.048 (1.002-1.098)	4.50E-02	1.059 (1.003-1.117)	3.70E-02	1.057 (0.990-1.129)	9.40E-02	0.12
rs17069904	0.935 (0.878-1.001)	5.22E-02	0.918 (0.849-0.993)	3.30E-02	0.887 (0.805-0.979)	1.70E-02	0.002
rs884205	1.048 (0.995-1.098)	6.23E-02	1.063 (1.004-1.125)	3.60E-02	1.066 (0.996-1.141)	6.70E-02	1

## Supplementary Information

Supplementary information, Figure S1 Generation of *K5Cre;Brca1;p53* double and *K5Cre;Rank;Brca1;p53* triple knockout mice.

Supplementary information, Figure S2 Cre-mediated deletion efficiency in *K5Cre* and *WapCre^C^* mice using a *Rosa26^eYFP^* reporter line.

Supplementary information, Figure S3 *Rank* deletion in basal mammary epithelial cells reduces *Brca1;p53* mutation-driven hyperplastic proliferation.

Supplementary information, Figure S4 DNA damage and epithelial origin of *WapCre^C^;Brca1;p53* and *WapCre^C^;Rank;Brca1;p53* tumors.

Supplementary information, Figure S5 Generation of *WapCre^C^;Brca1;p53* double and *WapCre^C^;Rank;Brca1;p53* triple knockout mice.

Supplementary information, Figure S6 Histopathologic assessment.

Supplementary information, Figure S7 Histological assessment of tumors from *WapCre^C^;Brca1;p53* and *WapCre^C^;Rank;Brca1;p53* mutant mice.

Supplementary information, Figure S8 Gene expression profiling of *WapCre^C^;Brca1;p53* and *WapCre^C^;Rank;Brca1;p53* tumors.

Supplementary information, Figure S9 Detection of proliferation and DNA damage in *WapCre^C^;Brca1;p53* and *WapCre^C^;Rank;Brca1;p53* mammary tumors.

Supplementary information, Figure S10 Analysis of a tumor free, 2 year old *WapCre^C^;Rank;Brca1;p53* triple mutant female.

Supplementary information, Figure S11 Pharmacological RANKL inhibition prevents the development of pre-neoplastic regions.

Supplementary information, Figure S12 Analysis of murine mammary progenitor cells.

Supplementary information, Table S1 RANKL and RANK protein expression in human breast tumors irrespective of *BRCA* status.

Supplementary information, Table S2 Genotyped iCOGS variants and breast cancer association in *BRCA1* mutation carriers.

Supplementary information, Table S3 Genotyped iCOGS variants and breast cancer association in *BRCA2* mutation carriers.

## References

[bib1] Miki Y, Swensen J, Shattuck-Eidens D, et al. A strong candidate for the breast and ovarian cancer susceptibility gene BRCA1. Science 1994; 266:66–71. 7545954 10.1126/science.7545954

[bib2] Widschwendter M, Rosenthal AN, Philpott S, et al. The sex hormone system in carriers of BRCA1/2 mutations: a case-control study. Lancet Oncol 2013; 14:1226–1232. 24140203 10.1016/S1470-2045(13)70448-0

[bib3] Poole AJ, Li Y, Kim Y, Lin SC, Lee WH, Lee EY. Prevention of Brca1-mediated mammary tumorigenesis in mice by a progesterone antagonist. Science 2006; 314:1467–1470. 17138902 10.1126/science.1130471

[bib4] Dougall WC, Glaccum M, Charrier K, et al. RANK is essential for osteoclast and lymph node development. Genes Dev 1999; 13:2412–2424. 10500098 10.1101/gad.13.18.2412 PMC317030

[bib5] Kong YY, Yoshida H, Sarosi I, et al. OPGL is a key regulator of osteoclastogenesis, lymphocyte development and lymph-node organogenesis. Nature 1999; 397:315–323. 9950424 10.1038/16852

[bib6] Cummings SR, San Martin J, McClung MR, et al. Denosumab for prevention of fractures in postmenopausal women with osteoporosis. N Engl J Med 2009; 361:756–765. 19671655 10.1056/NEJMoa0809493

[bib7] McClung MR, Lewiecki EM, Cohen SB, et al. Denosumab in postmenopausal women with low bone mineral density. N Engl J Med 2006; 354:821–831. 16495394 10.1056/NEJMoa044459

[bib8] Smith MR, Egerdie B, Hernandez Toriz N, et al. Denosumab in men receiving androgen-deprivation therapy for prostate cancer. N Engl J Med 2009; 361:745–755. 19671656 10.1056/NEJMoa0809003 PMC3038121

[bib9] Schramek D, Sigl V, Penninger JM. RANKL and RANK in sex hormone-induced breast cancer and breast cancer metastasis. Trends Endocrinol Metab 2011; 22:188–194. 21470874 10.1016/j.tem.2011.02.007

[bib10] Fata JE, Kong YY, Li J, et al. The osteoclast differentiation factor osteoprotegerin-ligand is essential for mammary gland development. Cell 2000; 103:41–50. 11051546 10.1016/s0092-8674(00)00103-3

[bib11] Joshi PA, Jackson HW, Beristain AG, et al. Progesterone induces adult mammary stem cell expansion. Nature 2010; 465:803–807. 20445538 10.1038/nature09091

[bib12] Asselin-Labat ML, Vaillant F, Sheridan JM, et al. Control of mammary stem cell function by steroid hormone signalling. Nature 2010; 465:798–802. 20383121 10.1038/nature09027

[bib13] Schramek D, Leibbrandt A, Sigl V, et al. Osteoclast differentiation factor RANKL controls development of progestin-driven mammary cancer. Nature 2010; 468:98–102. 20881962 10.1038/nature09387 PMC3084017

[bib14] Gonzalez-Suarez E, Jacob AP, Jones J, et al. RANK ligand mediates progestin-induced mammary epithelial proliferation and carcinogenesis. Nature 2010; 468:103–107. 20881963 10.1038/nature09495

[bib15] Liu S, Ginestier C, Charafe-Jauffret E, et al. BRCA1 regulates human mammary stem/progenitor cell fate. Proc Natl Acad Sci USA 2008; 105:1680–1685. 18230721 10.1073/pnas.0711613105 PMC2234204

[bib16] Hanada R, Leibbrandt A, Hanada T, et al. Central control of fever and female body temperature by RANKL/RANK. Nature 2009; 462:505–509. 19940926 10.1038/nature08596

[bib17] Berton TR, Matsumoto T, Page A, et al. Tumor formation in mice with conditional inactivation of Brca1 in epithelial tissues. Oncogene 2003; 22:5415–5426. 12934101 10.1038/sj.onc.1206825

[bib18] Liu X, Holstege H, van der Gulden H, et al. Somatic loss of BRCA1 and p53 in mice induces mammary tumors with features of human BRCA1-mutated basal-like breast cancer. Proc Natl Acad Sci USA 2007; 104:12111–12116. 17626182 10.1073/pnas.0702969104 PMC1924557

[bib19] Lin SC, Lee KF, Nikitin AY, et al. Somatic mutation of p53 leads to estrogen receptor alpha-positive and -negative mouse mammary tumors with high frequency of metastasis. Cancer Res 2004; 64:3525–3532. 15150107 10.1158/0008-5472.CAN-03-3524

[bib20] Pfefferle AD, Herschkowitz JI, Usary J, et al. Transcriptomic classification of genetically engineered mouse models of breast cancer identifies human subtype counterparts. Genome Biol 2013; 14:R125. 24220145 10.1186/gb-2013-14-11-r125 PMC4053990

[bib21] Couch FJ, Wang X, McGuffog L, et al. Genome-wide association study in BRCA1 mutation carriers identifies novel loci associated with breast and ovarian cancer risk. PLoS Genet 2013; 9:e1003212. 23544013 10.1371/journal.pgen.1003212 PMC3609646

[bib22] Gaudet MM, Kuchenbaecker KB, Vijai J, et al. Identification of a BRCA2-specific modifier locus at 6p24 related to breast cancer risk. PLoS Genet 2013; 9:e1003173. 23544012 10.1371/journal.pgen.1003173 PMC3609647

[bib23] Estrada K, Styrkarsdottir U, Evangelou E, et al. Genome-wide meta-analysis identifies 56 bone mineral density loci and reveals 14 loci associated with risk of fracture. Nat Genet 2012; 44:491–501. 22504420 10.1038/ng.2249 PMC3338864

[bib24] Rivadeneira F, Styrkarsdottir U, Estrada K, et al. Genetic Factors for Osteoporosis (GEFOS) Consortium, Twenty bone-mineral-density loci identified by large-scale meta-analysis of genome-wide association studies. Nat Genet 2009; 41:1199–1206. 19801982 10.1038/ng.446 PMC2783489

[bib25] Cancer Genome Atlas N. Comprehensive molecular portraits of human breast tumours. Nature 2012; 490:61–70. 23000897 10.1038/nature11412 PMC3465532

[bib26] Cordero A, Pellegrini P, Sanz-Moreno A, et al. Rankl impairs lactogenic differentiation through inhibition of the prolactin/Stat5 pathway at midgestation. Stem Cells 2016; 34:1027–1039. 26695351 10.1002/stem.2271

[bib27] Lee HJ, Gallego-Ortega D, Ledger A, et al. Progesterone drives mammary secretory differentiation via RankL-mediated induction of Elf5 in luminal progenitor cells. Development 2013; 140:1397–1401. 23462470 10.1242/dev.088948

[bib28] Obr AE, Grimm SL, Bishop KA, Pike JW, Lydon JP, Edwards DP. Progesterone receptor and Stat5 signaling cross talk through RANKL in mammary epithelial cells. Mol Endocrinol 2013; 27:1808–1824. 24014651 10.1210/me.2013-1077 PMC3805851

[bib29] Fan S, Wang J, Yuan R, Rosen EM, et al. BRCA1 inhibition of estrogen receptor signaling in transfected cells. Science 1999; 284:1354–1356. 10334989 10.1126/science.284.5418.1354

[bib30] Ma Y, Katiyar P, Jones LP, et al. The breast cancer susceptibility gene BRCA1 regulates progesterone receptor signaling in mammary epithelial cells. Mol Endocrinol 2006; 20:14–34. 16109739 10.1210/me.2004-0488 PMC4031608

[bib31] Jones LP, Li M, Halama ED, et al. Promotion of mammary cancer development by tamoxifen in a mouse model of Brca1-mutation-related breast cancer. Oncogene 2005; 24:3554–3562. 15750629 10.1038/sj.onc.1208426

[bib32] Dvinge H, Git A, Graf S, et al. The shaping and functional consequences of the microRNA landscape in breast cancer. Nature 2013; 497:378–382. 23644459 10.1038/nature12108

[bib33] Srinivas S, Watanabe T, Lin CS, et al. Cre reporter strains produced by targeted insertion of EYFP and ECFP into the ROSA26 locus. BMC Dev Biol 2001; 1:4. 11299042 10.1186/1471-213X-1-4 PMC31338

[bib34] Pencik J, Schlederer M, Gruber W, et al. STAT3 regulated ARF expression suppresses prostate cancer metastasis. Nat Commun 2015; 6:7736. 26198641 10.1038/ncomms8736 PMC4525303

[bib35] Rudmann D, Cardiff R, Chouinard L, et al. Proliferative and nonproliferative lesions of the rat and mouse mammary, Zymbal's, preputial, and clitoral glands. Toxicol Pathol 2012; 40:7S–39S. 10.1177/0192623312454242 22949413

[bib36] Cardiff RD, Anver MR, Gusterson BA, et al. The mammary pathology of genetically engineered mice: the consensus report and recommendations from the Annapolis meeting. Oncogene 2000; 19:968–988. 10713680 10.1038/sj.onc.1203277

[bib37] Frech MS, Halama ED, Tilli MT, et al. Deregulated estrogen receptor a expression in mammary epithelial cells of transgenic mice results in the development of ductal carcinoma *in situ*. Cancer Res 2005; 65:681–685. 15705859 PMC6952528

[bib38] Eirew P, Stingl J, Raouf A, et al. A method for quantifying normal human mammary epithelial stem cells with *in vivo* regenerative ability. Nat Med 2008; 14:1384–1389. 19029987 10.1038/nm.1791

[bib39] Stingl J, Emerman JT, Eaves CJ. Enzymatic dissociation and culture of normal human mammary tissue to detect progenitor activity. Methods Mol Biol 2005; 290:249–263. 15361667 10.1385/1-59259-838-2:249

[bib40] Chen X, Iliopoulos D, Zhang Q, et al. XBP1 promotes triple-negative breast cancer by controlling the HIF1alpha pathway. Nature 2014; 508:103–107. 24670641 10.1038/nature13119 PMC4105133

[bib41] Kim S, You S, Hwang D. Aminoacyl-tRNA synthetases and tumorigenesis: more than housekeeping. Nat Rev Cancer 2011; 11:708–718. 21941282 10.1038/nrc3124

[bib42] Joshi PA, Waterhouse PD, Kannan N, et al. RANK signaling amplifies WNT-responsive mammary progenitors through R-SPONDIN1. Stem Cell Reports 2015; 5:31–44. 26095608 10.1016/j.stemcr.2015.05.012 PMC4618445

[bib43] Herschkowitz JI, Simin K, Weigman VJ, et al. Identification of conserved gene expression features between murine mammary carcinoma models and human breast tumors. Genome Biol 2007; 8:R76. 17493263 10.1186/gb-2007-8-5-r76 PMC1929138

[bib44] Leek JT, Johnson WE, Parker HS, Jaffe AE, Storey JD. The sva package for removing batch effects and other unwanted variation in high-throughput experiments. Bioinformatics 2012; 28:882–883. 22257669 10.1093/bioinformatics/bts034 PMC3307112

[bib45] Chen X, Li J, Gray WH, et al. TNBCtype: a subtyping tool for triple-negative breast cancer. Cancer Inform 2012; 11:147–156. 22872785 10.4137/CIN.S9983 PMC3412597

[bib46] Lehmann BD, Bauer JA, Chen X, et al. Identification of human triple-negative breast cancer subtypes and preclinical models for selection of targeted therapies. J Clin Invest 2011; 121:2750–2767. 21633166 10.1172/JCI45014 PMC3127435

