# Supplementary material for: RANKL/RANK control Brca1 mutation-driven mammary tumors
Source: Cell Res. 2016 May 31;26(7):761–74. doi: 10.1038/cr.2016.69 (PMC5129883; doi:10.1038/cr.2016.69)
Supplement: Supplementary information, Figure S1 — Generation of K5Cre;Brca1;p53 double and K5Cre;Rank;Brca1;p53 triple knockout mice. [file cr201669x1.pdf]

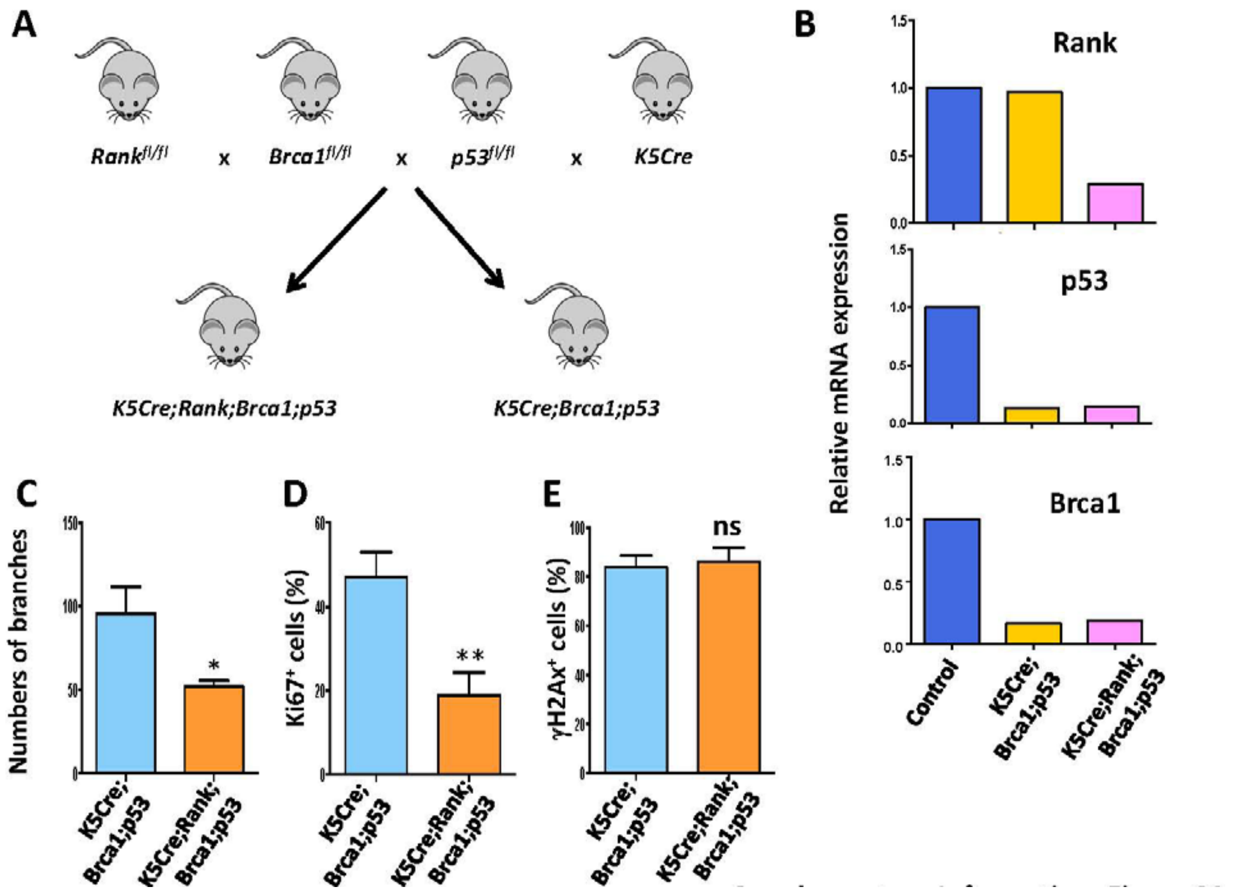

Supplementary information, Figure S1. Generation of *K5Cre;Brca1;p53* double and *K5Cre;Rank;Brca1;p53* triple knockout mice.

(**A**) Breeding scheme for the generation of *K5Cre;Brca1;p53* double and littermate *K5Cre;Rank;Brca1;p53* triple knockout mice. (**B**) Relative mRNA expression levels of *Rank* and *p53* in *K5Cre;Brca1;p53* double and *K5Cre;Rank;Brca1;p53* triple knockout mice. Expression levels of *Rank*, *Brca1*, and *p53* mRNA were determined using purified mammary epithelial cells by qRT-PCR. β-actin mRNA was used for normalization. Data are shown as fold change compared to Cre-negative littermate controls carrying all three respective floxed alleles. (**C**) Quantification of branching points in mammary glands of 4 month old *K5Cre;Brca1;p53* double and *K5Cre;Rank;Brca1;p53* triple knockout mice. Data represent the average number of branching points in five randomly selected areas +/- SEM. * $p<0.05$ (Student's t-test); n=5mice/group. (**D**)

and (**E**)**,** Quantification of Ki67$^+$ (**D**) and γH2Ax$^+$ (**E**) epithelial cells in mammary glands of *K5Cre;Rank;Brca1;p53* triple compared to *K5Cre;Brca1;p53* double mutant mice at 4 months of age. Data represent the average percentage of Ki67$^+$ and γH2Ax$^+$ cells per total mammary epithelial cells +/- SEM. ** $p < 0.005$; ns, not significant (Student's t-test; n=5 mice/group).
