# Supplementary material for: RANKL/RANK control Brca1 mutation-driven mammary tumors
Source: Cell Res. 2016 May 31;26(7):761–74. doi: 10.1038/cr.2016.69 (PMC5129883; doi:10.1038/cr.2016.69)
Supplement: Supplementary information, Figure S2 — Cre-mediated deletion efficiency in K5Cre and WapCreC mice using a Rosa26eYFP reporter line. [file cr201669x2.pdf]

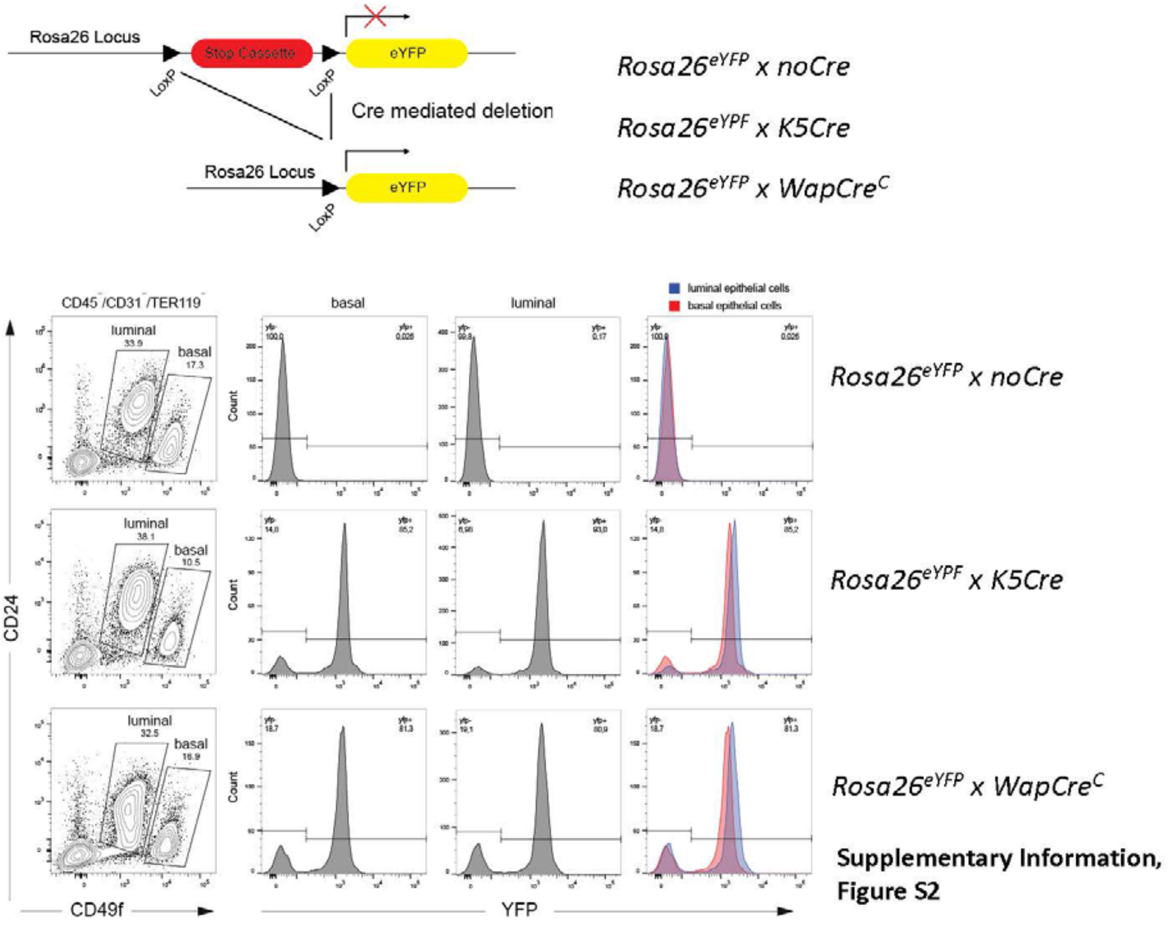

Supplementary information, Figure S2. Cre-mediated deletion efficiency in *K5Cre* and *WapCre*^{C} mice using a *Rosa26*^{eYFP} reporter line.

(**A**) Schema showing Cre-mediated deletion using the *Rosa26*^{eYPF} reporter mouse line. (**B**) Representative FACS blots and histograms of *Rosa26*^{eYFP} control (no Cre), *Rosa26*^{eYPF} *x K5Cre* and *Rosa26*^{eYFP} *x WapCre*^{C} mice showing Cre-mediated deletion in basal as well luminal epithelial cells.
