# Supplementary material for: RANKL/RANK control Brca1 mutation-driven mammary tumors
Source: Cell Res. 2016 May 31;26(7):761–74. doi: 10.1038/cr.2016.69 (PMC5129883; doi:10.1038/cr.2016.69)
Supplement: Supplementary information, Figure S3 — Rank deletion in basal mammary epithelial cells reduces Brca1;p53 mutation-driven hyperplastic proliferation. [file cr201669x3.pdf]

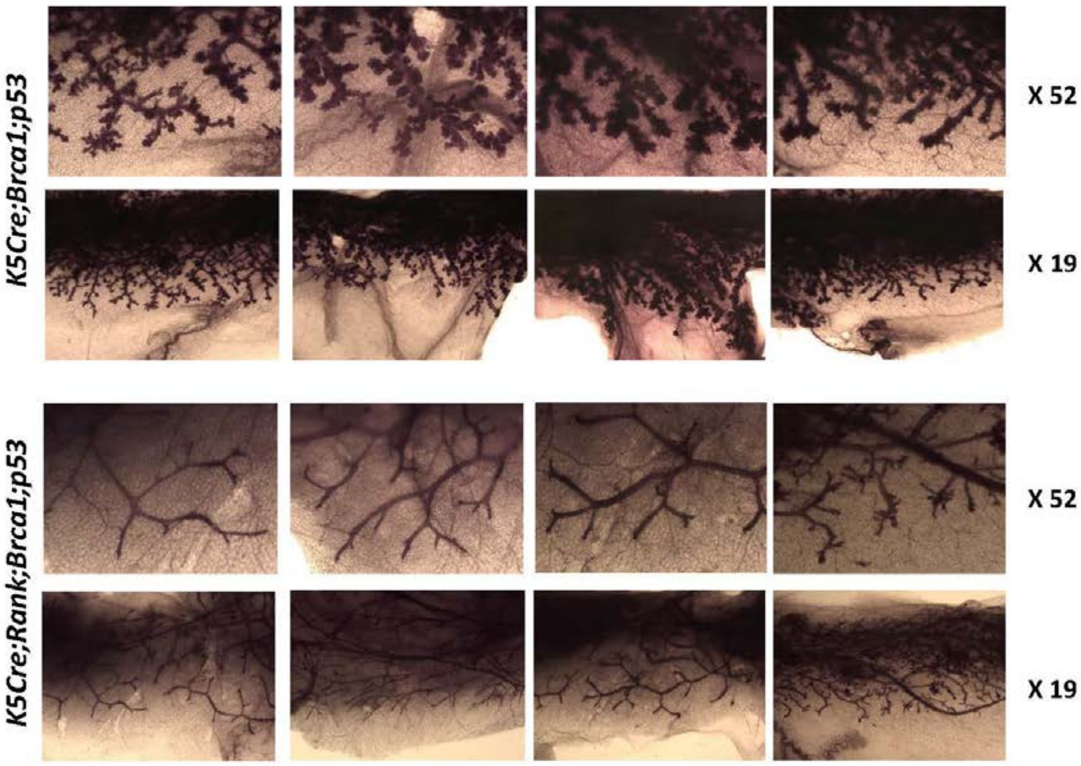

Supplementary information, Figure S3. *Rank* deletion in basal mammary epithelial cells reduces *Brca1;p53* mutation-driven hyperplastic proliferation.

Representative whole mount images of individual *K5Cre;Brca1;p53* double and littermate *K5Cre;Rank;Brca1;p53* triple knockout mice at 4 months of age. Data for 4 different mice are shown for each genotype. Magnifications are indicated.
