# Supplementary material for: RANKL/RANK control Brca1 mutation-driven mammary tumors
Source: Cell Res. 2016 May 31;26(7):761–74. doi: 10.1038/cr.2016.69 (PMC5129883; doi:10.1038/cr.2016.69)
Supplement: Supplementary information, Figure S4 — DNA damage and epithelial origin of WapCreC;Brca1;p53 and WapCreC;Rank;Brca1;p53 tumors. [file cr201669x4.pdf]

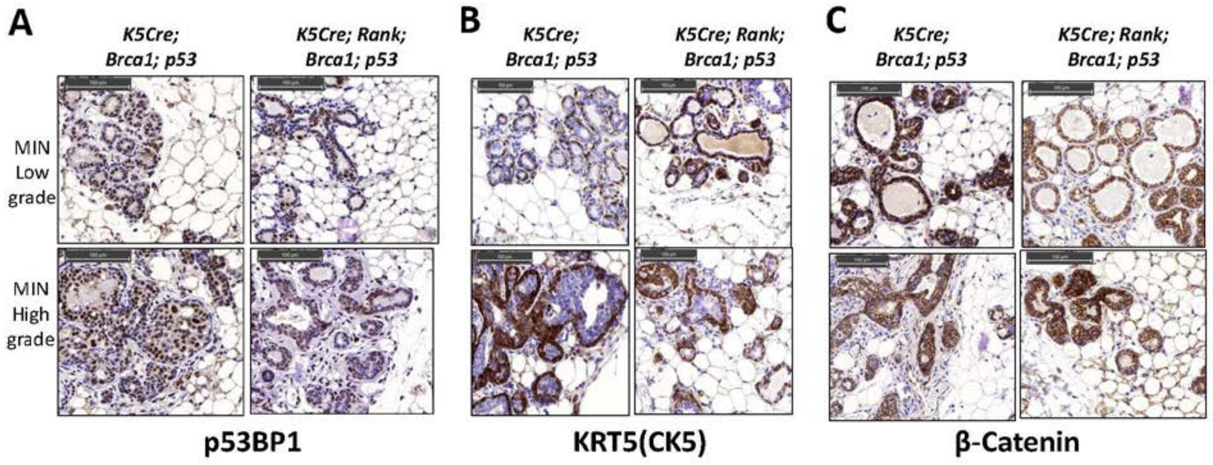

Supplementary information, Figure S4. DNA damage and epithelial origin of *WapCre^{C};Brca1;p53* and *WapCre^{C};Rank;Brca1;p53* tumors.

Representative (**A**) p53BP1, (**B**) Cytokeratin 5 (KTR5/CK5), and **c,** β-catenin (CTNNB1) immunostaining of MIN low grad and MIN high grade mammary tumors from *K5Cre;Brca1;p53* double and littermate *K5Cre;Rank;Brca1;p53* triple knockout mice at 4 months of age. Scale bars are indicated.
