# Supplementary material for: RANKL/RANK control Brca1 mutation-driven mammary tumors
Source: Cell Res. 2016 May 31;26(7):761–74. doi: 10.1038/cr.2016.69 (PMC5129883; doi:10.1038/cr.2016.69)
Supplement: Supplementary information, Figure S5 — Generation of WapCreC;Brca1;p53 double and WapCreC;Rank;Brca1;p53 triple knockout mice. [file cr201669x5.pdf]

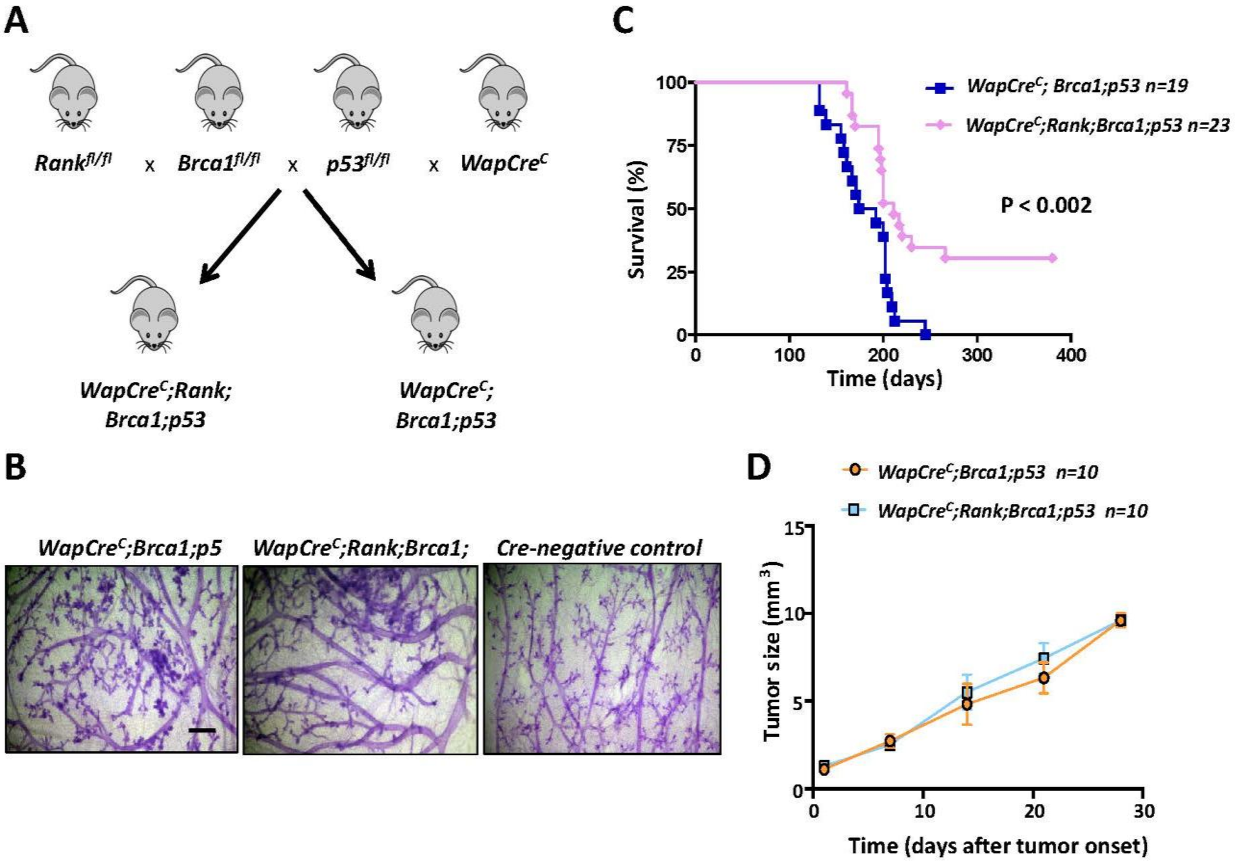

Supplementary information, Figure S5. Generation of *WapCre^C;Brca1;p53* double and *WapCre^C;Rank;Brca1;p53* triple knockout mice.

(**A**) Breeding scheme to generate *WapCre^{C};Brca1;p53* double and *WapCre^{C};Rank;Brca1;p53* triple knockout mice. (**B**) Representative whole mounts (Hematoxylin stain) from 3-4 month old *WapCre^{C};Brca1;p53* double knockout, *WapCre^{C};Rank;Brca1;p53* triple knockout and control mice that carry all three floxed alleles but were Cre-negative, demonstrating apparently normal mammary gland development during puberty. Scale bar represents 500µm. (**C**) Overall survival rates in *WapCre^{C};Brca1;p53* and *WapCre^{C};Rank;Brca1;p53* mice. Data are shown as percentage of overall mouse survival. (**D**) Tumor growth rates were comparable between *WapCre^{C};Brca1;p53* double and littermate *WapCre^{C};Rank;Brca1;p53* triple knockout mice. Data are shown as average tumor volume (mm^3) +/- SEM. Mice were sacrificed for ethical reasons when tumors reached a volume of $10mm^3$.
