# Supplementary material for: RANKL/RANK control Brca1 mutation-driven mammary tumors
Source: Cell Res. 2016 May 31;26(7):761–74. doi: 10.1038/cr.2016.69 (PMC5129883; doi:10.1038/cr.2016.69)
Supplement: Supplementary information, Figure S6 — Histopathologic assessment. [file cr201669x6.pdf]

A

Mouse	Genotype	Histopathology Grading (morphologic assessment and enumeration - *Pannoramic Viewer*)					Tumor grade $_{TG}$
		Number of mitoses $_{NM}$	Mitotic activity score $_{SM}$	Glandular (tubular/acinar) differentiation score $_{SG}$	Nuclear pleomorphism (anisokaryosis) score $_{SN}$	Total score	
701/2	*WapCre*^{C}; *Brca1;p53* (DKO)	2	1	2	3	6	Intermediate
742/9		4	2	3	2	7	Intermediate
802/3		3	1	4	3	8	High
814/10		9	3	4	3	10	High
ncn/5		10	3	4	3	9	High
737/6	*WapCre*^{C}; *Rank*; *Brca1;p53* (TKO)	3	1	3	2	6	Intermediate
737/9		6	2	3	2	6	Intermediate
745/2		6	2	4	3	6	Intermediate
748/6		2	1	4	3	6	Intermediate
810/9		8	2	3	3	6	Intermediate

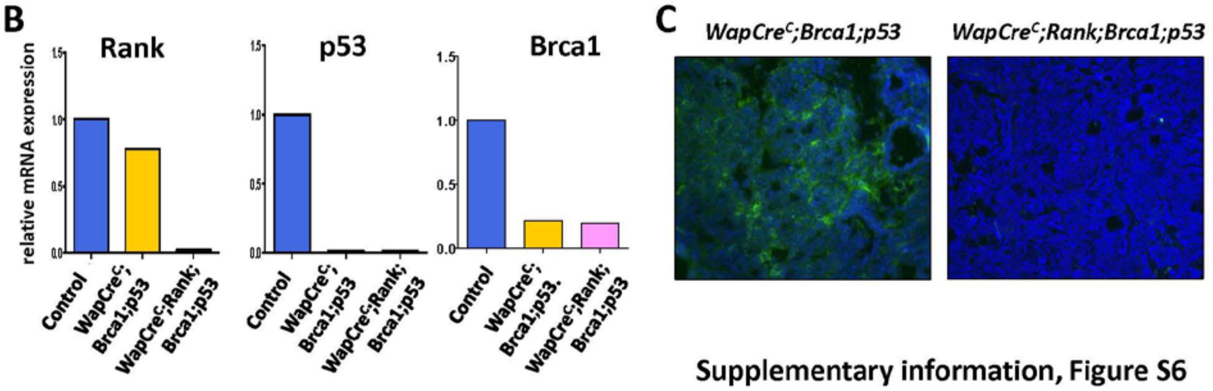

Supplementary information, Figure S6

Supplementary information, Figure S6. Histopathologic assessment.

(**A**) Semi-quantitative morphologic assessment and grading of primary mammary tumors derived from *WapCre*^{C}*;Brca1;p53* double knockout (DKO) and *WapCre*^{C}*;Rank;Brca1;p53* triple knockout (TKO) mice. Assessment was performed by certified pathologists. NM, Number of mitotic figures average count rounded off to the nearest integer from ten 40x digital fields within each tumor section. SM; Score representing the extent of mitotic activity; Scores: None = 0; 1 to 3 = low = 1; 4 to 8 = intermediate = 2, >8 = High = 3; SG, Score representing the degree of glandular differentiation indicated by the evidence of tubular and / or acinar structures with distinct lumina; Scores: None = 4; Low = 3, Intermediate = 2, High = 1; Well-differentiated and well-organized tubular and acinar structures = 0 (average value from ten 40x digital fields from each tumor

section). SN, Score representing the degree of nuclear pleomorphism (anisokaryosis) within tumor cells in a 40x digital field; Scores: Uniform nuclei = 0; Low = 1, Intermediate = 2, High = 3 (average value from ten 40x digital fields within each tumor section). TG, Low grade tumor = total score is less than or equal to 3; intermediate grade tumor = total score is greater than 3 but less than 8; high grade tumor = total score is equal to or greater than 8. (**B**) Relative mRNA expression levels of *Rank, Brca1,* and *p53* in *WapCre^{C};Brca1;p53* double and *WapCre^{C};Rank;Brca1;p53* triple knockout mammary tumors. Expression was determined by qRT-PCR and shown as relative values compared to mRNA expression levels in the control mice. Controls were mammary epithelial cells isolated from Cre- animals carrying the respective floxed alleles. (**C**) Anti-RANK immunofluorescence staining of mammary tumors derived from *WapCre^{C};Brca1;p53* and *WapCre^{C};Rank;Brca1;p53* knockout mice confirming *Rank* deletion in *WapCre^{C};Rank;Brca1;p53* tumors. Data are from late stage tumors, taken from mice once the tumor volume reached $10mm^3$.
