# Supplementary material for: RANKL/RANK control Brca1 mutation-driven mammary tumors
Source: Cell Res. 2016 May 31;26(7):761–74. doi: 10.1038/cr.2016.69 (PMC5129883; doi:10.1038/cr.2016.69)
Supplement: Supplementary information, Figure S7 — Histological assessment of tumors from WapCreC;Brca1;p53 and WapCreC;Rank;Brca1;p53 mutant mice. [file cr201669x7.pdf]

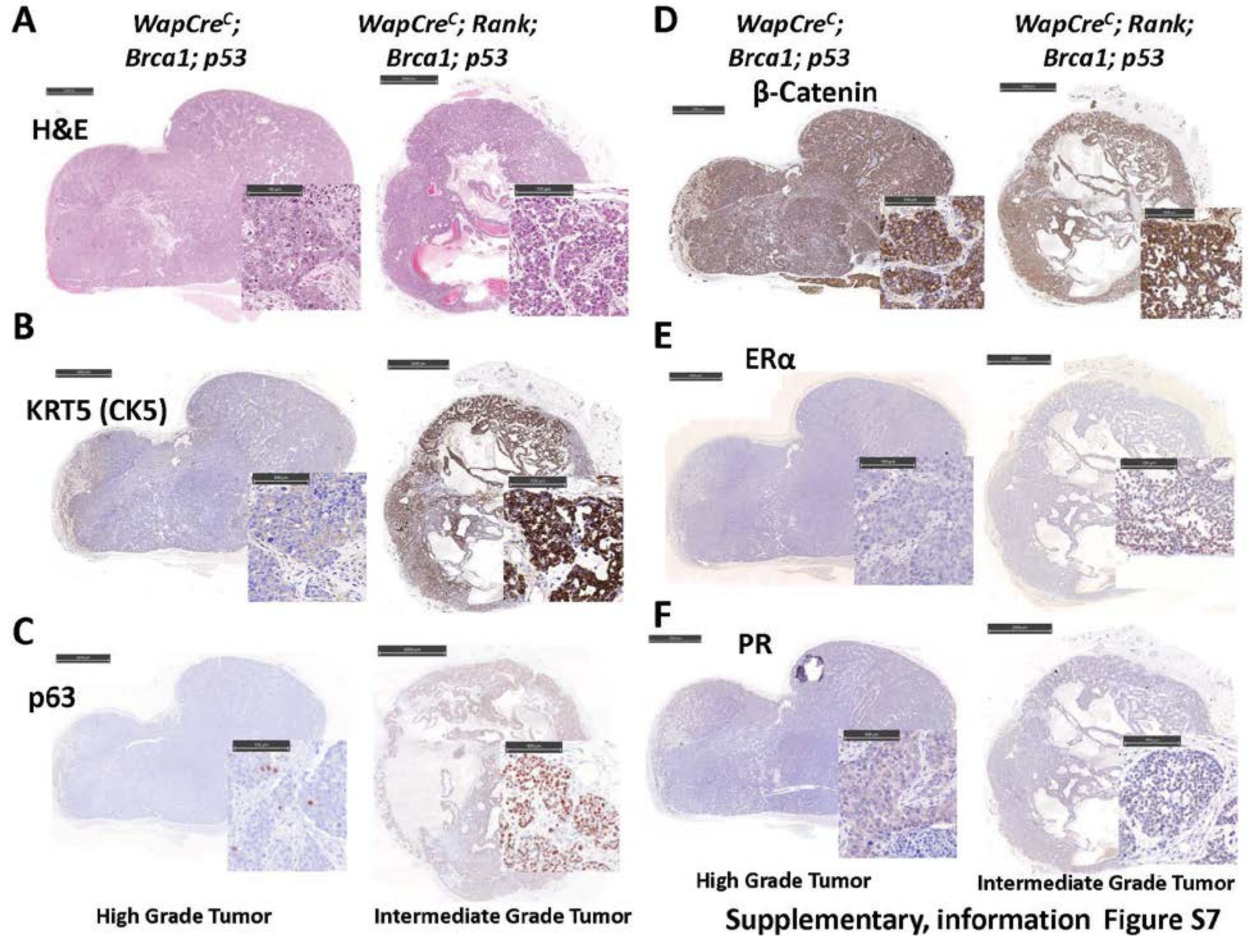

Supplementary information, Figure S7. Histological assessment of tumors from *WapCre^{C};Brca1;p53* and *WapCre^{C};Rank;Brca1;p53* mutant mice.

Representative (**A**) H&E stains, and (**B**) Cytokeratin 5 (KTR5/CK5), (**C**) p63, (**D**) β-catenin (CTNNB1), (**E**) estrogen receptor α (ERα), and (**F**) Progesterone receptor (PR) immunostaining of intermediate grade mammary tumors from *WapCre^{C};Rank;Brca1;p53* triple knockout mice and high grad mammary tumors from littermate *WapCre^{C};Brca1;p53* double knockout females. Inserts show higher magnifications of the same tumors. Scale bars are indicated.
