# Supplementary material for: RANKL/RANK control Brca1 mutation-driven mammary tumors
Source: Cell Res. 2016 May 31;26(7):761–74. doi: 10.1038/cr.2016.69 (PMC5129883; doi:10.1038/cr.2016.69)
Supplement: Supplementary information, Figure S8 — Gene expression profiling of WapCreC;Brca1;p53 and WapCreC;Rank;Brca1;p53 tumors. [file cr201669x8.pdf]

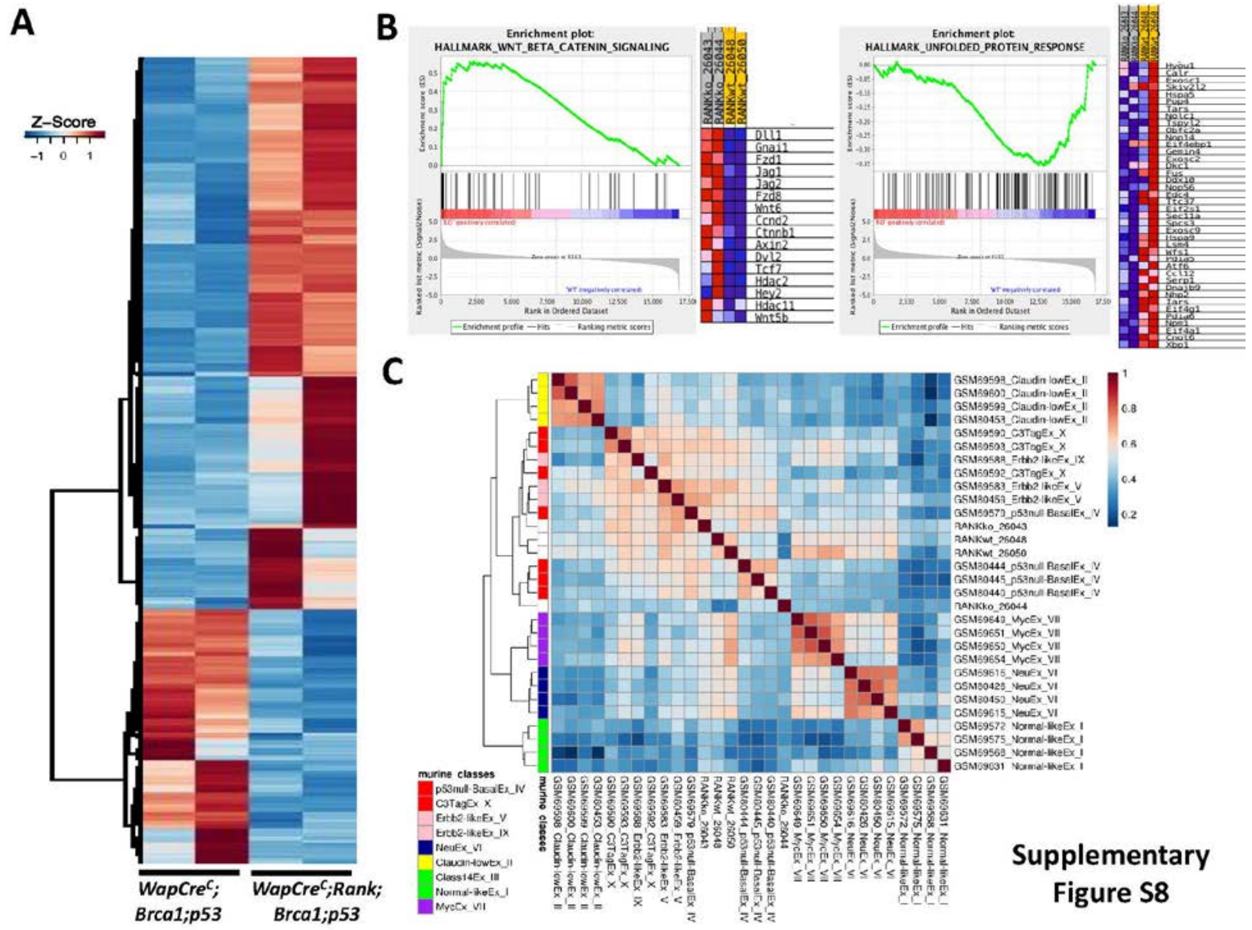

Supplementary information, Figure S8. Gene expression profiling of *WapCre^C;Brca1;p53* and *WapCre^C;Rank;Brca1;p53* tumors.

(**A**) Heat map of significantly differentially expressed genes (DeSeq2; 5% FDR) between *WapCre^C;Brca1;p53* and *WapCre^C;Rank;Brca1;p53* mammary tumor samples showing scaled expression values, with the minimum expression value for each gene in blue and the maximum value in red. Data are from late stage tumors, taken from mice once the tumor volume reached $10mm^3$. (**B**) Gene sets enriched in late stage ($10mm^3$) mammary tumors from *WapCre^C;Brca1;p53* and *WapCre^C;Rank;Brca1;p53* mice using GSEA (normalized enrichment score (NES). Left, GSEA enrichment plot of the enrichment score (ES; y-axis) reflecting the degree of the gene sets overrepresentation in *WapCre^C;Rank;Brca1;p53* or *WapCre^C;Brca1;p53* at the extreme left or

right of the entire ranked list. Solid bars represent genes of the gene set. Right, GSEA-derived heatmap illustrating gene expression profiles of the leading edge subset. Only two examples are shown, the complete data set has been deposited to GEO accession number GSE71362. (**C**) Heatmap showing Spearman correlations between our expression data in *WapCre*^{C}*;Rank;Brca1;p53* mammary tumor samples and published expression data from mouse breast cancer models[15,21]. Comparison shows samples for murine subclasses proposed to be conserved between human and mouse[15]. Coloring is based on the suggested similarity to human breast cancer subtypes as reported in[15]. Samples are listed with their NCBI GEO accession number, followed by the Herschkowitz et al. and Pfefferle et al. classification connected by an underscore (assignment as in ref 15). Sample subtypes are highlighted by the annotation bar left of the matrix. Coloring of subtypes indicates the determined human expression-based subtype counterparts: Basal-like (red), Claudin-low (yellow), HER2-enriched (pink), LuminalA (darkblue), Normal-like (green), basal-like and luminal B (purple).
