# Supplementary material for: RANKL/RANK control Brca1 mutation-driven mammary tumors
Source: Cell Res. 2016 May 31;26(7):761–74. doi: 10.1038/cr.2016.69 (PMC5129883; doi:10.1038/cr.2016.69)
Supplement: Supplementary information, Figure S9 — Detection of proliferation and DNA damage in WapCreC;Brca1;p53 and WapCreC;Rank;Brca1;p53 mammary tumors. [file cr201669x9.pdf]

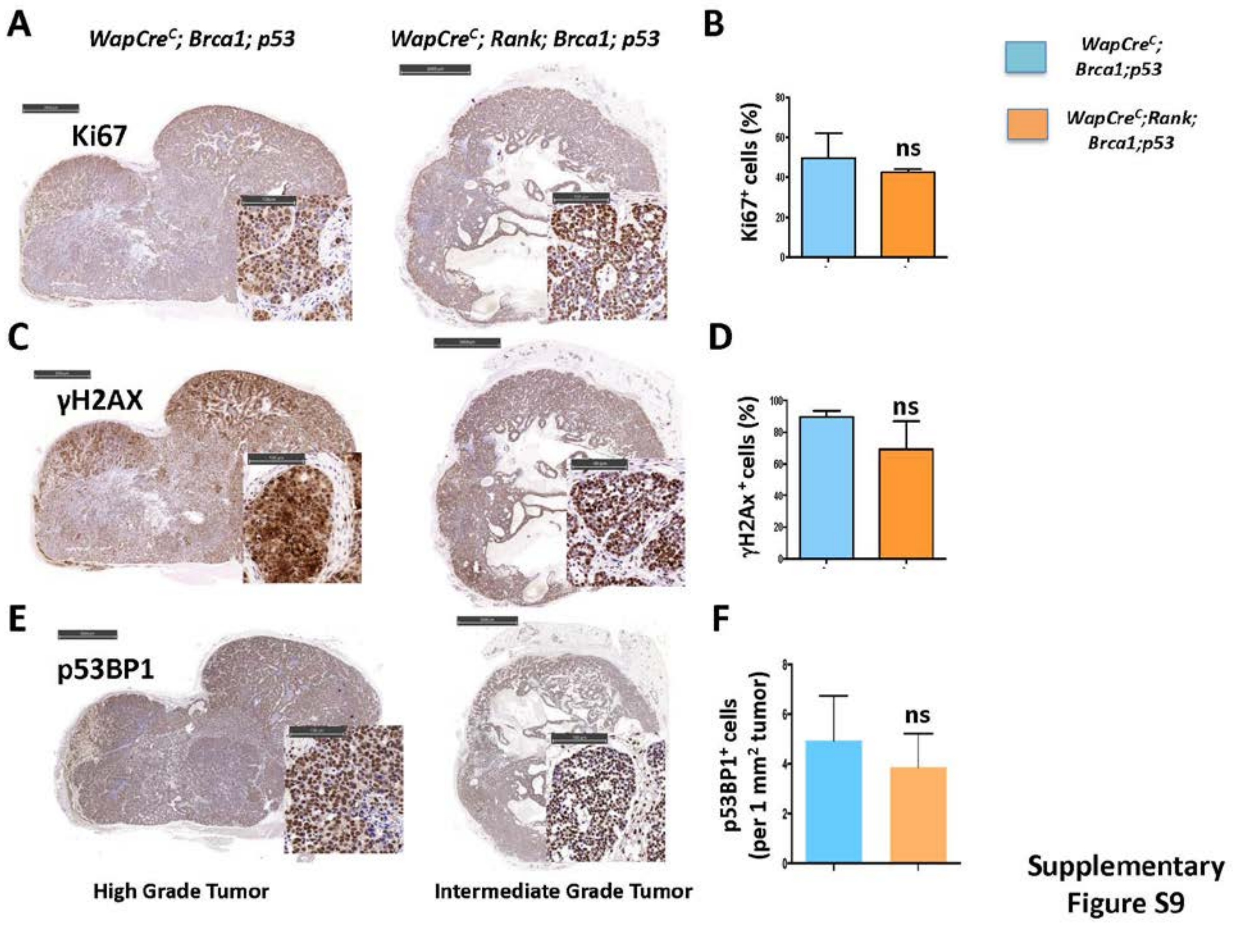

Supplementary information, Figure S9. Detection of proliferation and DNA damage in *WapCre^C;Brca1;p53* and *WapCre^C;Rank;Brca1;p53* mammary tumors.

Representative images and quantifications of (**A**), (**B**), Ki67, (**C**), (**D**), γH2Ax, and (**E**), (**F**), p53BP1 immunostaining of intermediate grade mammary tumors from *WapCre^C;Rank;Brca1;p53* triple knockout mice and high grad mammary tumors from littermate *WapCre^C;Brca1;p53* double knockout females. Inserts show higher magnifications of the same tumors. Scale bars are shown. Data in (**B**) and (**D**) shown as mean percentages of $Ki67^+$ and $γH2Ax^+$ cells; +/- SEM; n=5 mice/group. ns, not significant; Student's t-Test. Data in (**F**) are shown as p53BP1 positive cells per 100 $μm^2$ of tumour tissue.
