# Supplementary material for: RANKL/RANK control Brca1 mutation-driven mammary tumors
Source: Cell Res. 2016 May 31;26(7):761–74. doi: 10.1038/cr.2016.69 (PMC5129883; doi:10.1038/cr.2016.69)
Supplement: Supplementary information, Figure S10 — Analysis of a tumor free, 2 year old WapCreC;Rank;Brca1;p53 triple mutant female. [file cr201669x10.pdf]

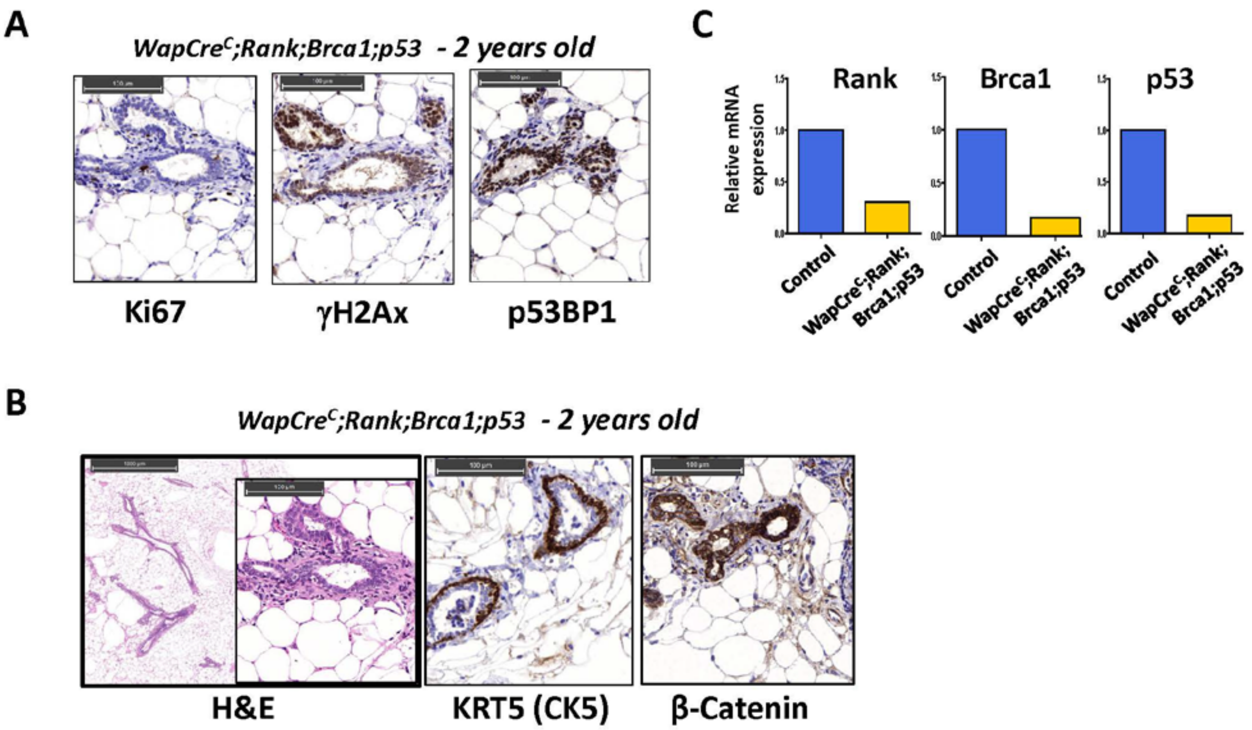

Supplementary information, Figure S10. Analysis of a tumor free, 2 year old *WapCre^{C};Rank;Brca1;p53* triple mutant female.

(**A**) H&E stains and KRT5 (CK5) and β-catenin (CTNNB1) immunohistochemistry of mammary glands from a 2 year old tumor free *WapCre^{C};Rank;Brca1;p53* mutant mouse, showing apparently normal mammary gland morphology. Scale bars are indicated. (**B**) Ki67, γH2Ax, and p53BP1 immunostainings of mammary glands from a 2 year old, tumor free *WapCre^{C};Rank;Brca1;p53* triple mutant female, showing low Ki67 positivity and marked DNA damage as determined by γH2Ax and p53BP1 positivity**.** Scale bars are indicated. (**C**) Relative mRNA expression levels of *Rank*, *p53* and *Brca1* in mammary epithelial cells (MECs) isolated from a 2 year old tumor free *WapCre^{C};Rank;Brca1;p53* triple knockout mouse. Expression levels of *Rank*, *p53* and *Brca1* mRNA were determined by qRT-PCR. β-actin mRNA was used for normalization. Data are shown as relative expression levels as compared to Cre-negative littermate controls carrying the respective floxed alleles.
