# Supplementary material for: RANKL/RANK control Brca1 mutation-driven mammary tumors
Source: Cell Res. 2016 May 31;26(7):761–74. doi: 10.1038/cr.2016.69 (PMC5129883; doi:10.1038/cr.2016.69)
Supplement: Supplementary information, Figure S11 — Pharmacological RANKL inhibition prevents the development of pre-neoplastic regions. [file cr201669x11.pdf]

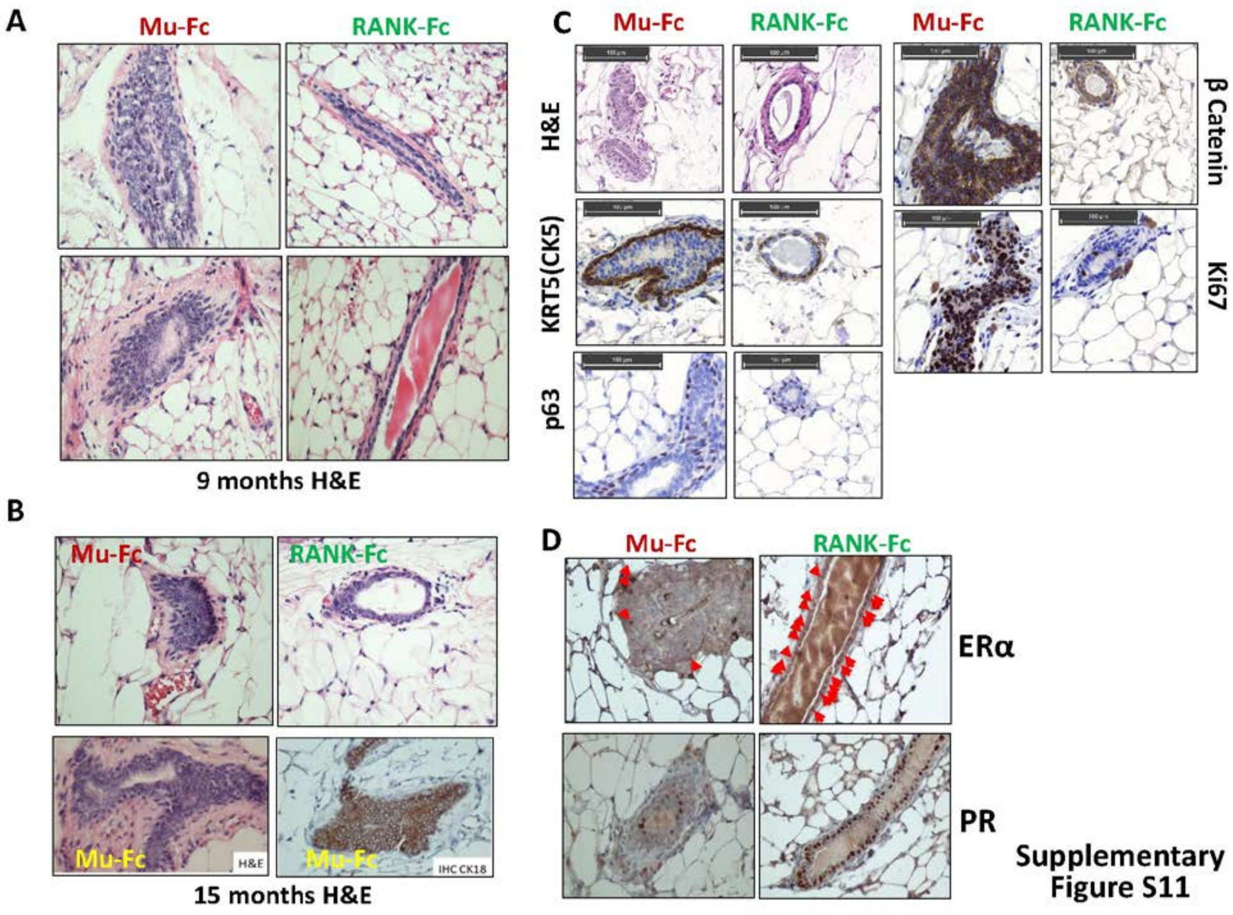

Supplementary information, Figure S11. Pharmacological RANKL inhibition prevents the development of pre-neoplastic regions.

(**A**) Representative H&E stained sections of mammary tissue of *MMTV-CreBrca1*^{flox11} mice that received Mu-Fc or RANK-Fc and were sacrificed at 9 month of age. (**B**) Representative H&E stained section (left panel) and immunohistochemistry (IHC) detection of Cytokeratin 18 (CK18) of inguinal mammary glands from Mu-Fc-treated *MMTV-CreBrca1*^{flox11} mice at 15 months of age. (**C**) Representative H&E stains, and Cytokeratin 5 (KTR5/CK5), p63, β-catenin (CTNNB1), and Ki67 immunostaining of inguinal mammary glands from Mu-Fc or RANK-Fc treated *MMTV-CreBrca1*^{flox11} mice at 15 months of age. Scale bars are indicated. (**D**) Immunohistochemical detection of estrogen receptor-α (ERα) and progesterone receptor (PR) of inguinal mammary

glands from *MMTV-Cre Brca1*$^{flox11/flox11}$ mice at 9 months of age treated with Mu-Fc or RANK-Fc. Red arrows indicate representative cells demonstrating ERα expression. Of note, when all mammary epithelial cells in the visual fields were counted, we observed statistically significant differences in the percentage of mammary epithelium that stained positive for nuclear ER α between the mu-Fc RANK-Fc treated female *MMTV-Cre Brca1*$^{flox11/flox11}$ mice at both 9 and 15 month time points (9 months: 18.1 ± 2.1 vs. 10.1± 4.1 mean ± SD; 15 months: 26.9 ± 5.1 vs. 15.1± 7.0, Rank-Fc vs. mu-Fc). Additionally, we observed increased PR expression in mammary epithelial cells of RANK-Fc treated mice (9 months: 40.6 ± 8.6 vs. 22.0±8.7 mean±SD, RANK-Fc vs. Mu-Fc; 15 months: 47.1 ± 7.5 vs. 29.0± 7.0, RANK-Fc vs. Mu-Fc). All Magnifications x 400.
