# Supplementary material for: RANKL/RANK control Brca1 mutation-driven mammary tumors
Source: Cell Res. 2016 May 31;26(7):761–74. doi: 10.1038/cr.2016.69 (PMC5129883; doi:10.1038/cr.2016.69)
Supplement: Supplementary information, Figure S12 — Analysis of murine mammary progenitor cells. [file cr201669x12.pdf]

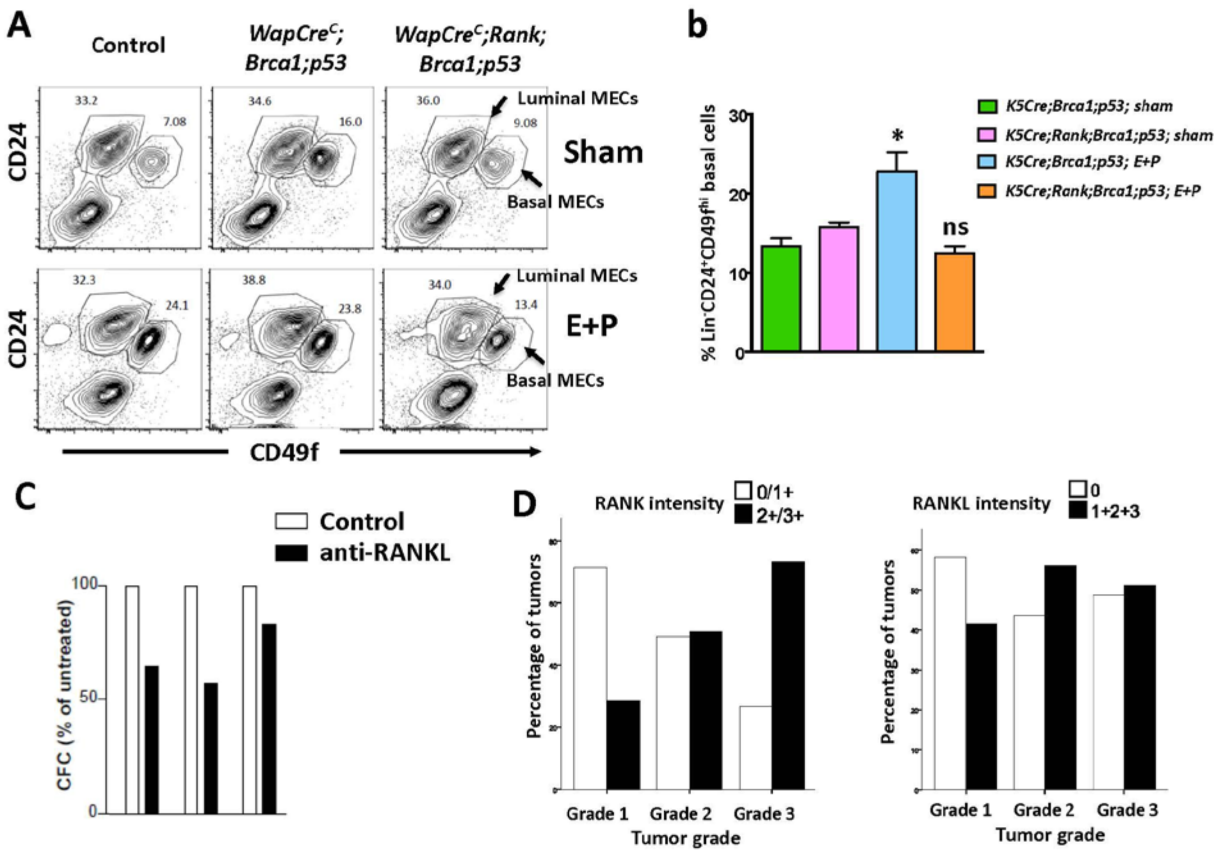

Supplementary information, Figure S12. Analysis of murine mammary progenitor cells.

(**A**) Representative FACS profiles of luminal ($CD24^{+}CD49f^{lo}$) and basal ($CD24^{+}CD49f^{hi}$) mammary epithelial cell populations (MECs) in ovariectomized WapCre^{C}-negative control, *WapCre^{C};Brca1;p53* double knockout and *WapCre^{C};Rank:Brca1;p53* triple knockout female mice treated with 17β-estradiol plus progesterone (E+P) or left untreated (sham). (**B**) Impaired expansion of $Lin^{-}CD24^{+}CD49f^{hi}$ basal mammary epithelial cells in 10 weeks old ovariectomized *K5Cre;Rank;Brca1;p53* mice in response to 17β-estradiol and progesterone (E+P). Sham treated (no hormones) *K5Cre;Brca1;p53* and *K5Cre;Rank;Brca1;53* littermates are shown as controls. Data represent mean +/- SEM of at least n=3 mice/group. * $p<0.05$; ns, not significant; Student's t-Test comparing the respective sham to E+P treated cohorts. (**C**) Colony forming capacity of

human mammary progenitor epithelial cells isolated from three women who carry heterozygous *BRCA1* mutations. Single mammary cell preparations were generated from organoids for CFC assays, plated, and left either untreated or were treated with the anti-RANKL blocking Ab Denosumab (1μg/ml). Data for all three individuals are shown as percentages of CFCs compared to the untreated controls (100%). (**D**) Percentages of low grade 1, intermediate grade2 and high grade 3 human breast tumors (irrespective of their *BRCA* mutation status) that expresses no/low (0/1) or high (2/3) RANK and RANKL. RANK and RANKL protein expression were determined by immunohistochemistry and staining intensity evaluated by certified pathologists. For detailed patient samples see Supplementary Table 1.
