# Supplementary material for: RANKL/RANK control Brca1 mutation-driven mammary tumors
Source: Cell Res. 2016 May 31;26(7):761–74. doi: 10.1038/cr.2016.69 (PMC5129883; doi:10.1038/cr.2016.69)
Supplement: Supplementary information, Table S1 — RANKL and RANK protein expression in human breast tumors irrespective of BRCA status. [file cr201669x13.pdf]

Supplementary Table 1. RANKL and RANK protein expression in human breast tumors irrespective of *BRCA* status.

	RANK Intensity[2]					RANKL Intensity[2]				
	0/1+ (N=111)		2+/3+ (N=137)			0 (N=111)		1+/2+ (N=131)		
	N	%	N	%	*P*	N	%	N	%	*P*
Tumor grade					<0.001					0.471
I	15	71.4	6	28.6		14	58.3	10	41.7	
II	33	49.3	34	50.7		28	43.8	36	56.3	
III	23	26.7	63	73.3		41	48.8	43	51.2	
Unknown[1]	40	54.1	34	45.9		28	40.0	42	60.0	
ER					0.059					0.548
Negative	34	37.8	56	62.2		47	51.6	44	48.4	
Positive	63	50.8	61	49.2		56	47.5	62	52.5	
Unknown[1]	14	41.2	20	58.8		8	24.2	25	75.8	
PR					0.009					0.776
Negative	42	37.8	69	62.2		54	50.0	54	50.0	
Positive	59	55.7	47	44.3		49	48.0	53	52.0	
Unknown[1]	10	32.3	21	67.7		8	25.0	24	75.0	
HER2					0.003					0.694
Negative	66	53.7	57	46.3		66	54.1	56	45.9	
Positive	18	30.5	41	69.5		28	50.9	27	49.1	
Unknown[1]	27	40.9	39	59.1		17	26.2	48	73.8	

[1] Excluded from analyses. [2] Row percentages presented. *p*-values (Chi-square or Fisher's Exact test). RANK and RANKL expression were determined by immunohistochemistry by certified pathologists.
