# Supplementary material for: RANKL/RANK control Brca1 mutation-driven mammary tumors
Source: Cell Res. 2016 May 31;26(7):761–74. doi: 10.1038/cr.2016.69 (PMC5129883; doi:10.1038/cr.2016.69)
Supplement: Supplementary information, Table S2 — Genotyped iCOGS variants and breast cancer association in BRCA1 mutation carriers. [file cr201669x14.pdf]

Supplementary Table 2. Genotyped iCOGS variants and breast cancer association in *BRCA1* mutation carriers.

SNP ID	Chromosome	Position (bp)	Gene	Allele 1	Allele 2	Breast cancer unaffected (n)	Breast cancer unaffected (MAF)	Breast cancer affected (n)	Breast cancer affected (MAF)	Breast cancer log HR	P value
rs9646629	18	58202179	*TNFRSF11A*	G	C	7453	0,351	7797	0,362	0,051	2,15E-02
rs4485469	18	58150553	*TNFRSF11A*	A	G	7454	0,455	7797	0,443	-0,045	3,55E-02
rs34739845	18	58175415	*TNFRSF11A*	A	G	7455	0,118	7796	0,112	-0,069	4,07E-02
rs4941129	18	58151457	*TNFRSF11A*	A	G	7432	0,290	7770	0,300	0,047	4,50E-02
rs17069904	18	58183929	*TNFRSF11A*	G	A	7455	0,110	7796	0,105	-0,067	5,22E-02
rs884205	18	58205837	*TNFRSF11A*	C	A	7454	0,232	7796	0,239	0,047	6,23E-02
c18_pos58205737	18	58205737	*TNFRSF11A*	A	G	7454	0,101	7792	0,096	-0,063	8,10E-02
rs2980968	18	58219105	*TNFRSF11A*	G	A	7369	0,098	7681	0,104	0,056	1,15E-01
rs7235803	18	58151359	*TNFRSF11A*	A	G	7437	0,326	7779	0,332	0,034	1,30E-01
rs4369774	18	58161428	*TNFRSF11A*	C	A	7454	0,457	7795	0,465	0,032	1,37E-01
rs3018352	18	58225845	*TNFRSF11A*	C	A	7449	0,098	7795	0,104	0,053	1,38E-01
rs2957146	18	58228274	*TNFRSF11A*	A	G	7455	0,098	7796	0,104	0,053	1,39E-01
rs6567273	18	58184023	*TNFRSF11A*	A	T	7420	0,466	7747	0,460	-0,032	1,40E-01
rs8086340	18	58157958	*TNFRSF11A*	C	G	7451	0,453	7793	0,461	0,030	1,55E-01
rs2980971	18	58225044	*TNFRSF11A*	G	A	7454	0,098	7796	0,104	0,050	1,56E-01
rs7239667	18	58180218	*TNFRSF11A*	C	G	7451	0,319	7796	0,328	0,031	1,70E-01
rs12959396	18	58190289	*TNFRSF11A*	A	C	7455	0,472	7797	0,465	-0,029	1,74E-01
rs8083511	18	58179635	*TNFRSF11A*	A	C	7423	0,187	7769	0,195	0,037	1,81E-01
c18_pos58203566	18	58203566	*TNFRSF11A*	C	A	7380	0,000	7733	0,000	-2,286	2,08E-01
rs8083014	18	58181567	*TNFRSF11A*	C	A	7455	0,236	7796	0,243	0,030	2,38E-01
c18_pos58188348	18	58188348	*TNFRSF11A*	A	G	7450	0,034	7793	0,035	0,067	2,59E-01
c18_pos58155239	18	58155239	*TNFRSF11A*	A	G	7455	0,076	7797	0,074	-0,045	2,71E-01
rs6567266	18	58145613	*TNFRSF11A*	G	A	7455	0,199	7796	0,195	-0,029	2,85E-01
rs7226420	18	58211525	*TNFRSF11A*	G	A	7455	0,270	7797	0,268	-0,025	3,00E-01
rs17069898	18	58180261	*TNFRSF11A*	A	G	7454	0,378	7797	0,384	0,023	3,03E-01
rs8095109	18	58208116	*TNFRSF11A*	A	G	7455	0,295	7795	0,292	-0,024	3,09E-01
c18_pos58143977	18	58143977	*TNFRSF11A*	G	A	7433	0,247	7770	0,253	0,025	3,13E-01
rs12165104	18	58201621	*TNFRSF11A*	G	A	7454	0,273	7797	0,271	-0,024	3,25E-01
rs8089829	18	58182884	*TNFRSF11A*	A	G	7412	0,465	7750	0,464	-0,021	3,40E-01
rs17665435	18	58208321	*TNFRSF11A*	T	A	7448	0,332	7794	0,328	-0,021	3,48E-01
rs4500848	18	58165443	*TNFRSF11A*	G	A	7455	0,060	7797	0,062	0,041	3,60E-01
rs9948182	18	58198469	*TNFRSF11A*	G	A	7454	0,332	7795	0,330	-0,018	4,31E-01
rs4627473	18	58212143	*TNFRSF11A*	A	C	7451	0,076	7794	0,075	-0,031	4,40E-01
rs12954567	18	58197184	*TNFRSF11A*	G	A	7455	0,046	7797	0,046	0,035	4,90E-01
rs6567280	18	58214602	*TNFRSF11A*	A	G	7453	0,404	7795	0,402	-0,013	5,37E-01
rs7226991	18	58139671	*TNFRSF11A*	G	A	7453	0,296	7795	0,294	-0,012	6,21E-01
rs17069906	18	58199374	*TNFRSF11A*	A	G	7455	0,025	7796	0,026	0,030	6,56E-01
rs4940552	18	58207928	*TNFRSF11A*	A	C	7455	0,112	7796	0,113	0,013	6,98E-01
rs34256674	18	58180197	*TNFRSF11A*	A	G	7455	0,487	7797	0,486	-0,008	6,99E-01
rs9960450	18	58167855	*TNFRSF11A*	A	G	7455	0,048	7797	0,048	0,019	7,02E-01
rs4072376	18	58121563	*TNFRSF11A*	G	A	7454	0,167	7797	0,166	-0,008	7,83E-01
rs2957125	18	58209322	*TNFRSF11A*	T	A	7454	0,423	7796	0,421	0,006	7,94E-01
rs11874087	18	58162686	*TNFRSF11A*	G	A	7454	0,026	7797	0,026	-0,016	8,05E-01
rs8092336	18	58187063	*TNFRSF11A*	G	A	7455	0,057	7796	0,057	-0,010	8,34E-01
c18_pos58147995	18	58147995	*TNFRSF11A*	G	A	7454	0,058	7797	0,057	0,009	8,44E-01
rs2156209	18	58215516	*TNFRSF11A*	G	A	7453	0,061	7796	0,061	0,008	8,55E-01
c18_pos58147700	18	58147700	*TNFRSF11A*	G	A	7455	0,068	7797	0,069	-0,006	8,80E-01
rs1805034	18	58178221	*TNFRSF11A*	A	G	7455	0,483	7797	0,481	0,003	8,87E-01
c18_pos58205057	18	58205057	*TNFRSF11A*	G	C	7455	0,101	7796	0,101	-0,003	9,41E-01
rs2939421	18	58110971	*TNFRSF11A*	A	G	7452	0,306	7797	0,304	-0,002	9,42E-01
rs7236029	18	58208041	*TNFRSF11A*	A	G	7455	0,069	7796	0,068	-0,001	9,88E-01
