# Supplementary material for: RANKL/RANK control Brca1 mutation-driven mammary tumors
Source: Cell Res. 2016 May 31;26(7):761–74. doi: 10.1038/cr.2016.69 (PMC5129883; doi:10.1038/cr.2016.69)
Supplement: Supplementary information, Table S3 — Genotyped iCOGS variants and breast cancer association in BRCA2 mutation carriers. [file cr201669x15.pdf]

Supplementary Table 3. Genotyped iCOGS variants and breast cancer association in *BRCA2* mutation carriers.

SNP ID	Chromosome	Position (bp)	Allele 1	Allele 2	Breast cancer unaffected (n)	Breast cancer unaffected (MAF)	Breast cancer affected (n)	Breast cancer affected (MAF)	Breast cancer log HR	P value
c18_pos58147700	18	58147700	G	A	3880	0,077	4330	0,065	-0,146	1,43E-02
rs9960450	18	58167855	A	G	3881	0,060	4330	0,050	-0,137	4,05E-02
rs4485469	18	58150553	A	G	3881	0,449	4330	0,435	-0,058	6,13E-02
rs1805034	18	58178221	A	G	3878	0,488	4326	0,476	-0,053	8,26E-02
rs12959396	18	58190289	A	C	3881	0,455	4330	0,473	0,051	9,70E-02
rs8089829	18	58182884	A	G	3861	0,464	4310	0,477	0,049	1,07E-01
rs11152342	18	58166187	G	C	3854	0,000	4304	0,000	-2,918	1,20E-01
c18_pos58143977	18	58143977	G	A	3875	0,246	4322	0,262	0,050	1,46E-01
rs8086340	18	58157958	C	G	3881	0,473	4328	0,456	-0,032	2,90E-01
rs17069906	18	58199374	A	G	3880	0,027	4329	0,029	0,098	2,95E-01
rs6567266	18	58145613	G	A	3881	0,199	4330	0,186	-0,040	3,01E-01
rs7226991	18	58139671	G	A	3879	0,295	4329	0,294	-0,033	3,15E-01
rs6567280	18	58214602	A	G	3879	0,419	4328	0,410	-0,030	3,30E-01
rs4369774	18	58161428	C	A	3880	0,476	4330	0,461	-0,029	3,43E-01
rs11874087	18	58162686	G	A	3881	0,026	4328	0,026	-0,090	3,50E-01
rs2957125	18	58209322	T	A	3881	0,401	4329	0,412	0,028	3,67E-01
rs7239667	18	58180218	C	G	3881	0,329	4330	0,330	0,029	3,73E-01
rs4941129	18	58151457	A	G	3879	0,294	4328	0,296	0,025	4,55E-01
rs8083511	18	58179635	A	C	3880	0,198	4328	0,199	0,026	4,93E-01
rs8092336	18	58187063	G	A	3881	0,056	4330	0,058	0,041	5,32E-01
rs4940552	18	58207928	A	C	3881	0,124	4330	0,117	-0,028	5,40E-01
rs12954567	18	58197184	G	A	3881	0,049	4329	0,046	-0,044	5,41E-01
rs7236029	18	58208041	A	G	3881	0,068	4330	0,064	-0,035	5,57E-01
rs8083014	18	58181567	C	A	3881	0,244	4329	0,246	0,020	5,67E-01
rs4072376	18	58121563	G	A	3881	0,170	4330	0,159	-0,023	5,74E-01
rs2156209	18	58215516	G	A	3881	0,059	4328	0,056	-0,032	6,18E-01
rs8095109	18	58208116	A	G	3881	0,298	4329	0,297	-0,016	6,34E-01
rs884205	18	58205837	C	A	3878	0,236	4329	0,238	0,016	6,67E-01
rs9646629	18	58202179	G	C	3879	0,359	4330	0,361	0,014	6,69E-01
rs34739845	18	58175415	A	G	3880	0,109	4330	0,107	-0,021	6,71E-01
rs2980968	18	58219105	G	A	3864	0,103	4299	0,104	0,020	6,96E-01
rs2980971	18	58225044	G	A	3881	0,103	4330	0,103	0,015	7,67E-01
rs17069904	18	58183929	G	A	3881	0,100	4329	0,103	0,014	7,76E-01
rs4627473	18	58212143	A	C	3879	0,078	4329	0,076	-0,015	7,85E-01
rs6567273	18	58184023	A	T	3875	0,461	4325	0,464	0,007	8,19E-01
rs2957146	18	58228274	A	G	3881	0,103	4330	0,103	0,011	8,25E-01
c18_pos58188348	18	58188348	A	G	3881	0,033	4330	0,034	0,018	8,33E-01
rs4500848	18	58165443	G	A	3880	0,060	4330	0,059	-0,012	8,47E-01
rs12165104	18	58201621	G	A	3881	0,274	4330	0,277	0,006	8,59E-01
rs7226420	18	58211525	G	A	3881	0,270	4330	0,274	0,006	8,61E-01
rs17069898	18	58180261	A	G	3881	0,398	4330	0,388	-0,004	8,87E-01
rs7235803	18	58151359	A	G	3879	0,333	4328	0,328	0,004	8,93E-01
c18_pos58147995	18	58147995	G	A	3880	0,053	4330	0,053	0,009	8,95E-01
rs34256674	18	58180197	G	A	3879	0,505	4329	0,510	-0,004	8,98E-01
rs9948182	18	58198469	G	A	3880	0,331	4327	0,334	0,003	9,34E-01
rs17665435	18	58208321	T	A	3876	0,330	4324	0,332	-0,002	9,46E-01
c18_pos58155239	18	58155239	A	G	3881	0,079	4330	0,079	0,002	9,74E-01
c18_pos58205737	18	58205737	A	G	3879	0,092	4328	0,095	0,002	9,74E-01
rs3018352	18	58225845	C	A	3880	0,104	4330	0,104	0,001	9,79E-01
c18_pos58205057	18	58205057	G	C	3880	0,104	4329	0,104	0,001	9,81E-01
rs2939421	18	58110971	A	G	3880	0,305	4330	0,300	0,001	9,83E-01
